# Functional Biology and Molecular Mechanisms of Host-Pathogen Interactions for Aflatoxin Contamination in Groundnut (*Arachis hypogaea* L.) and Maize (*Zea mays* L.)

**DOI:** 10.3389/fmicb.2020.00227

**Published:** 2020-03-03

**Authors:** Pooja Soni, Sunil S. Gangurde, Alejandro Ortega-Beltran, Rakesh Kumar, Sejal Parmar, Hari K. Sudini, Yong Lei, Xinzhi Ni, Dongxin Huai, Jake C. Fountain, Samuel Njoroge, George Mahuku, Thankappan Radhakrishnan, Weijian Zhuang, Baozhu Guo, Boshou Liao, Prashant Singam, Manish K. Pandey, Ranajit Bandyopadhyay, Rajeev K. Varshney

**Affiliations:** ^1^International Crops Research Institute for the Semi-Arid Tropics, Hyderabad, India; ^2^International Institute of Tropical Agriculture, Ibadan, Nigeria; ^3^Oil Crops Research Institute, Chinese Academy of Agricultural Sciences, Wuhan, China; ^4^Crop Genetics and Breeding Research Unit, United States Department of Agriculture – Agriculture Research Service, Tifton, GA, United States; ^5^Department of Plant Pathology, University of Georgia, Tifton, GA, United States; ^6^International Crops Research Institute for the Semi-Arid Tropics, Lilongwe, Malawi; ^7^International Institute of Tropical Agriculture, Dar es Salaam, Tanzania; ^8^Indian Council of Agricultural Research – Directorate of Groundnut Research, Junagadh, India; ^9^Oil Crops Research Institute, Fujian Agriculture and Forestry University, Fuzhou, China; ^10^Crop Protection and Management Research Unit, United States Department of Agriculture – Agricultural Research Service, Tifton, GA, United States; ^11^Department of Genetics, Osmania University, Hyderabad, India

**Keywords:** *Aspergillus flavus*, aflatoxin contamination, host-pathogen interactions, molecular mechanisms, QTLs, groundnut, maize

## Abstract

Aflatoxins are secondary metabolites produced by soilborne saprophytic fungus *Aspergillus flavus* and closely related species that infect several agricultural commodities including groundnut and maize. The consumption of contaminated commodities adversely affects the health of humans and livestock. Aflatoxin contamination also causes significant economic and financial losses to producers. Research efforts and significant progress have been made in the past three decades to understand the genetic behavior, molecular mechanisms, as well as the detailed biology of host-pathogen interactions. A range of omics approaches have facilitated better understanding of the resistance mechanisms and identified pathways involved during host-pathogen interactions. Most of such studies were however undertaken in groundnut and maize. Current efforts are geared toward harnessing knowledge on host-pathogen interactions and crop resistant factors that control aflatoxin contamination. This study provides a summary of the recent progress made in enhancing the understanding of the functional biology and molecular mechanisms associated with host-pathogen interactions during aflatoxin contamination in groundnut and maize.

## Introduction

Aflatoxins are teratogenic, carcinogenic and immunosuppressive secondary metabolites produced by several *Aspergillus* section Flavi species (Frisvad et al., 2019). The most common aflatoxin-producing species is *A. flavus* (Amaike and Keller, 2011) but, *A. parasiticus*, *A. nomius*, and other species may be important causal agents of contamination in some areas/years (Diedhiou et al., 2011; Probst et al., 2014; Kachapulula et al., 2017; Kumar P. et al., 2017). Aflatoxin-producing fungi contaminate several agricultural commodities such as groundnut, maize, cottonseed, wheat, rice, tree nuts, and chili peppers (Doster et al., 2014; Khan et al., 2014; Kumar P. et al., 2017; Sarma et al., 2017; Ezekiel et al., 2019).

Aflatoxin remains in food and feed even after cooking and drying of the crop because of its heat and freeze stable nature. There are four major types of aflatoxins, namely, AFB_1_, AFB_2_, AFG_1_, and AFG_2_ which are discernible based on their blue and green fluorescence under UV light and migration rate. AFB_1_, the most potent and toxic, is associated with hepatocellular carcinoma (Liu and Wu, 2010). Consuming contaminated commodities may have chronic and/or acute effects that may lead to mortality (Sarma et al., 2017). In addition to the large array of negative health effects of the toxins, the contamination of crops results in large economic losses to farmers and to countries because of produce rejected by markets seeking aflatoxin-compliant crops (Wild and Gong, 2010; Bryden, 2012). For instance, India could export only 800,000 tons each year despite being 2nd largest groundnut producer in the world, and aflatoxin contamination being one of the major reason behind low export (Suneja, 2019). In semi-arid and arid regions of the United States, and tropical and sub-tropical Asia and Africa, aflatoxin contamination of agricultural products occurs frequently (Cotty et al., 2008; Razzaghi-Abyanehed, 2013; Bandyopadhyay et al., 2016). In such affected areas, mitigation of contamination is necessary to protect the health of consumers, maintain crop competitiveness, and to harness the full potential of crops to ensure food and nutritional security.

Deploying pre- and post-harvest genetic resistance in new crop varieties together with good agricultural practices may provide a permanent solution to this problem (Ayalew et al., 2017; Meseka et al., 2018). In this context, it is imperative to explore and deploy all possible resistance mechanisms/methods to control aflatoxin accumulation in the field followed by best practices in the entire value chain. In the case of groundnut, three different types of resistance mechanisms, namely *in vitro* seed colonization (IVSC), pre-harvest aflatoxin contamination (PAC), and aflatoxin production (AP) have been reported, which are inherited independently (Nigam et al., 2009). In addition, genetic resistance is modulated by high soil temperature and moisture stress which promote higher rates of fungal infection and contamination. To achieve stable genetic resistance against *A. flavus* infection, we believe all three mechanisms should be examined and integrated to effectively provide resistance under field conditions, during harvest, and throughout storage (see Pandey et al., 2019).

Groundnut and maize are among the most aflatoxin-prone crops. Both are commonly exposed to *Aspergillus* infection during pre- and post-harvest stages (Guo et al., 2008). For example in Ghana, these two crops that are considered as staples are frequently infected by *Aspergillus* species, with unsafe aflatoxin levels (Samson et al., 1981; MoFA, 2011; Agbetiameh et al., 2018). In Ghana, as in any other country, aflatoxin-resistant varieties are not commercially available. In addition, farmers typically do not follow good agricultural practices; so contamination begins in the field and may continue until the crops are consumed. Therefore, farmers and traders must receive training and information on good agricultural practices such as timely sowing and irrigation, ensuring adequate dry field conditions before harvest, timely harvesting, and post-harvest management strategies to limit aflatoxin contamination (Dorner, 2004; Jaime-Garcia and Cotty, 2004; Hell et al., 2008; Florkowski and Kolavalli, 2013; Bandyopadhyay et al., 2016). Although some success has been achieved, good management practices are neither very cost effective nor always practical for the resource-poor farmers, or are not effective in reducing aflatoxin content below tolerance thresholds if not used as part of a holistic aflatoxin management strategy. Climate change and frequent extreme weather events, hot and dry conditions, and erratic rainfall have become more pronounced, allowing aflatoxin-producing fungi to thrive, exacerbating the frequency and severity of contamination events (Chen et al., 2015). Heat and drought stresses are the most important abiotic stresses that predispose crops to *Aspergillus* infection and also affect crop productivity.

A promising strategy is the field application of atoxigenic *A. flavus* strains to reduce aflatoxin content in crops. In the United States and several African countries, driven primarily by USDA-ARS and IITA, respectively, the application of carefully selected atoxigenic *A. flavus* strains as biocontrol agents has consistently reduced aflatoxin contamination in commercially produced crops and allowed farmers to enter domestic and international premium markets (Cotty et al., 2007; Dorner, 2009; Mehl et al., 2012; Doster et al., 2014; Bandyopadhyay et al., 2019; Ortega-Beltran and Bandyopadhyay, 2019; Schreurs et al., 2019; Senghor et al., 2019). When applied at the right stage, treated crops accumulate over 80% less and sometimes even 100% less aflatoxin than non-treated adjacent crops. In addition, when biocontrol is used as a centerpiece of a holistic aflatoxin management strategy, lower aflatoxins accumulate in treated crops at harvest and throughout storage (Bandyopadhyay et al., 2019). Research groups in Italy, Argentina, China, Thailand, and Australia have conducted extensive work on biocontrol in addition to the United States and Africa (Alaniz Zanon et al., 2013, 2016; Mauro et al., 2015; Pitt et al., 2015). Although significant progress has been made, there are many countries where the biocontrol technology has not yet been developed and in the meantime other aflatoxin management strategies need to be employed.

In rainfed areas where farmers are subjected to unavoidable biotic and abiotic stresses that influence aflatoxin accumulation, it is paramount to conduct comprehensive genetics and genomics studies for a better understanding of the genetic behavior, genetic architecture, and molecular mechanisms that govern different types of aflatoxin resistance in groundnut and maize. Several genetic mapping studies conducted in both groundnut and maize have concluded that aflatoxin resistance is a quantitative trait and has complex genetic behavior with high G × E interaction (Chen et al., 2015; Pandey et al., 2019). Hence, by dissecting host-pathogen interactions during fungal infection by aflatoxin producers and aflatoxin contamination, important host-specific, resistance-related genes/proteins/pathways/resistant factors can be characterized in both groundnut and maize. This study focusses on the current status of resistance and molecular mechanisms in these two major crops using different omics approaches such as genetics, genomics, transcriptomics, and proteomics in addition to emphasizing on host-pathogen interactions. We also discuss the research gaps in global efforts to understand resistance mechanisms and translational genomics in developing aflatoxin-resistant groundnut and maize varieties to provide safe products to consumers as well as safeguard the multibillion-dollar industries associated with both crops.

## General Characteristics of Aflatoxin-Producing Fungi

*Aspergillus* is a diverse genus of fungi that contains more than 200 species (Samson, 1992). Among those that produce aflatoxin, the agriculturally important species belong to section Flavi (Frisvad et al., 2019). Within section Flavi, *A. flavus* and *A. parasiticus* are the most common causal agents of aflatoxin contamination and are associated with a large number of crops (Pildain et al., 2008; Probst et al., 2014). *A. flavus* produces B aflatoxins and *A. parasiticus* produces both B and G aflatoxins. Some *A. flavus* strains cannot produce aflatoxin due to deletions or defects in the aflatoxin biosynthesis gene cluster (Chang et al., 2005; Adhikari et al., 2016). *A. flavus* strains may also produce other toxic compounds such as sterigmatocystin, cyclopiazonic acid, kojic acid, β-nitropropionic acid, aspertoxin, aflatrem, gliotoxin, and aspergillic acid (Hedayati et al., 2007); however, their incidence and frequency in field crops and toxicity to humans and animals are not clear.

Based on sclerotia size, *A. flavus* can be classified into two groups, L and S morphotypes. L morphotype produces few, large sclerotia (>400 μm), abundant conidia, and variable aflatoxin levels while S morphotype produces few conidia, abundant small sclerotia (<400 μm), and consistently high aflatoxin levels (Cotty, 1989). Some L morphotype strains do not produce aflatoxin due to lesions in the aflatoxin gene cluster and are known as atoxigenic (Chang et al., 2005; Adhikari et al., 2016). In nature, *A. flavus* produces primarily asexual spores (conidia) (Amaike and Keller, 2011). The fungus lives in the soil as conidia and the sclerotia, aggregates of hyphae that serve as survival structures that germinate to form saprophytically growing mycelia. Conidia are carried by wind or insects to host tissues, where they germinate and infect both aerial and subterraneanly grown organs of agronomically important crops (Cotty, 2001; Amaike and Keller, 2011); hence, insects may act as vectors during crop infection. Sclerotia allow aflatoxin producers to survive in extreme environmental conditions (Wicklow et al., 1993; Payne, 1998). Certain strains of *A. flavus* – both aflatoxin producers and atoxigenic strains – have higher adaptation and increased competitiveness in diverse cropping systems (Mehl and Cotty, 2011; Atehnkeng et al., 2016; Agbetiameh et al., 2019). Further, sexual reproduction has been reported to occur in *A. flavus*, *A. parasiticus*, and *A. nomius* under highly artificial laboratory conditions (Horn et al., 2009a, b) and also in the field after the release of *A. flavus* sclerotia incubated for 6 months (Horn et al., 2014). However, the significance of sexual reproduction in nature needs further studies.

## Factors Affecting Toxigenicity and Aflatoxin Contamination

Different biotic factors such as fungal virulence, host susceptibility, insect damage, and abiotic factors such as soil moisture, temperature, high humidity, and mechanical damage while attempting inter-cultivation practices significantly influence *A. flavus* invasion and aflatoxin accumulation in groundnut (Asis et al., 2005). In maize, hot and dry environments (>32°C and >70% RH), drought conditions and damage to kernel seed coat compromise predispose the crop to aflatoxin contamination. Under drought conditions, drought-tolerant varieties accumulate lower aflatoxin levels compared to non-drought-tolerant varieties. High grain moisture increases post-harvest molding and aflatoxin contamination. Hence, proper drying of grains after harvest to 7% moisture level in groundnut and 12% moisture level in maize is ideal to prevent fungal growth (Liang et al., 2009). Temperature is also an important factor as *A. flavus* thrives well in a wide range of temperatures between 10 and 40°C. However, the optimum temperature range for high AP by *A. flavus* is 25–30°C (Gqaleni et al., 1997). Storage conditions largely influence aflatoxin in crops. Storing pods/grains in jute bags provides favorable conditions for *A. flavus* growth. Jute bags can easily absorb moisture because of high porosity which favors rapid growth and multiplication of molds. Purdue Improved Crop Storage (PICS) bags that rely on the principle of hermetic storage have been used to prevent *A. flavus* infestation and aflatoxin contamination during storage (Sudini et al., 2015; Danso et al., 2018, 2019; Walker et al., 2018). Although aflatoxin contamination is more severe in the field during pre-harvest stage, contamination may increase during post-harvest if management practices such as transportation and storage are deficient. Hence, integrated management of aflatoxin contamination during pre-harvest, post-harvest and storage is necessary to reduce aflatoxin contamination and aflatoxin exposure.

## Genetics of Resistance Mechanisms

The mechanisms of resistance to infection and reduced AP are quantitative in nature (Warburton and Williams, 2014). In groundnut, the mechanisms include resistance to infection in the pod wall, resistance to seed invasion and colonization of seed coat, and resistance to AP in cotyledons. At the time of infection, aflatoxin producers have to penetrate the pod wall and then the seed coat to reach the cotyledons, from which they derive nutrients and produce aflatoxin. In groundnut, resistance to pod infection is attributed to pod shell structure, while resistance to seed invasion and colonization are mostly physical and attributed to seed coat thickness, density of palisade cell layers, and the presence of wax layers (Upadhyaya et al., 2002). In the case of maize, resistance mechanisms include good husk coverage, presence of proteins inhibiting fungal growth (Moore et al., 2004; Chen et al., 2010) wax, and cutin layers (Russin et al., 1997; Gembeh et al., 2001). Maize with kernel integrity intact and a living embryo typically accumulates less aflatoxin (Brown et al., 1993).

Generation mean analysis in maize has shown that additive and dominant gene action are important for resistance to AP (Campbell et al., 1997; Busboom and White, 2004). Diallele mating designs were used to study the inheritance of resistance to both *Aspergillus* ear rot and aflatoxin accumulation. These two studies reported that general combining ability had a greater effect on aflatoxin resistance in maize than specific combining ability, suggesting that additive gene effect is more important than dominant gene effect (Darrah et al., 1987; Gorman et al., 1992).

A resistant inbred of maize Oh516 was developed from the cross (B14 × L97) × B14 at Ohio State University and the hybrid derived from testcross Oh516 × B73 showed resistance to *A. flavus* infection and low aflatoxin concentration in grain (Campbell and White, 1995). The resistant inbred lines from testcross Oh516 × B73 were not significantly different from the inbred lines developed from the testcross Tex6 × B73 (Paul et al., 2003). F_1_ crosses developed with inbred lines Oh516 or Tex6 had lower aflatoxin concentration in grain than crosses without Oh516 or Tex6. The F_1_ cross Oh516 × Tex6 had the lowest aflatoxin concentration in grain of all F_1_ crosses. These findings indicate that the resistance mechanism is quantitative in nature and may be governed by multiple genes.

### Types of Resistance Mechanisms

**FIGURE 1:**
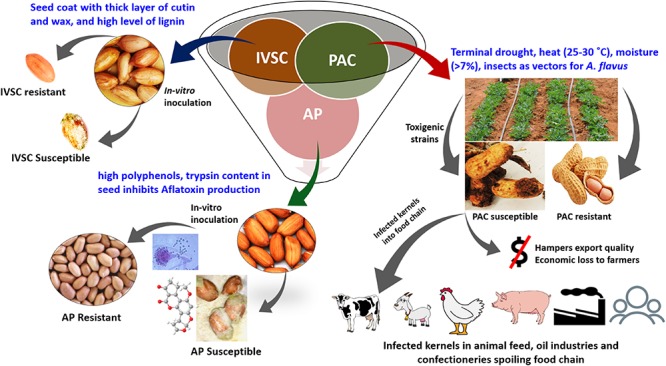
Aflatoxin resistance mechanisms in groundnut. IVSC, *in vitro* seed colonization; PAC, pre-harvest aflatoxin contamination; AP, aflatoxin production.

Groundnut has three types of resistance mechanisms, i.e., IVSC, PAC, and AP (Nigam et al., 2009; Figure 1). Similarly, in maize, the resistance is a sum of (1) prevention of fungal infection; (2) prevention of subsequent growth of the fungus after infection; and (3) inhibition of aflatoxin biosynthesis after infection (Williams et al., 2015). The extent of aflatoxin contamination varies with geographical location, cultural and agronomic practices, storage and processing period.

In groundnut, the majority of contamination occurs in the field. Hence in the context of developing aflatoxin-resistant groundnut cultivars, host resistance for PAC is a preventive approach that is economical and easy to disseminate. Such strategy does not require extra resources for farmers, leaves no chemical residues as a result of fungicide usage, and is an alternative for areas/nations where atoxigenic biocontrol measures are not available (Garrido-Bazan et al., 2018). ICRISAT has been deploying genetics and genomics approaches to understand resistance mechanisms and identify resistant genes/haplotypes to amalgamate all the three resistance mechanisms into a single genetic background in groundnut using genomics-assisted breeding (GAB) (Pandey et al., 2019). In addition to genetic resistance in groundnut and maize, reduced aflatoxin accumulation will require multidisciplinary approaches such as the use of biocontrol agents, good harvesting practices, appropriate drying, and optimal post-harvest storage (Logrieco et al., 2018). In the long run, the development of new breeding lines using introgression of validated quantitative trait loci (QTLs), single nucleotide polymorphism (SNPs) associated with resistance at the pre-harvest and/or post-harvest stages, optimized markers for marker-assisted selection (MAS), marker-assisted recurrent selection (MARS), and genomic selection (GS), can help the farming community grow crop varieties that may accumulate less/minimal aflatoxin.

### Physical and Chemical Barriers to Infection

In groundnut, seed coat thickness and its permeability confer resistance against *A. flavus* infection as a seed coat is the outermost layer that acts as a physical barrier (LaPrade et al., 1973). Smaller hila, a more compact arrangement of palisade-like layer of testa, and thicker waxy surface contribute to resistance against *A. flavus* infection (Taber et al., 1973). It has been reported that higher wax and cutin deposits in groundnut lead to resistance to *A. flavus* invasion and AP in resistant genotypes than in susceptible genotypes (Liang et al., 2003b). Hence, the seed coat, wax, and cutin are effective physical barriers to pathogen invasion and colonization. Groundnut testa is a rich source of tannins that inhibit *A. flavus* infection. 5-7-dimethoxyisoflavone (Turner et al., 1975) and tannins (Sanders and Mixon, 1979) have been reported as important inhibitors of *A. flavus* infection. In groundnut, tannins inhibit *A. parasiticus* growth by arresting mycelial growth and reducing AP (Sanders and Mixon, 1979). The basic composition of testa also contributes to the resistance to invasion. A study on protein profiling in a panel of 15 groundnut genotypes revealed that resistant genotypes had higher trypsin content and activity than susceptible genotypes (Liang et al., 2003a).

In maize, trypsin, ribosome-inactivating protein (RIP), and zeamatin act as inhibitors to the infection of *A. flavus* and *A. parasiticus*, and many other fungi (Chen et al., 1998). Resistance to colonization results from a variety of physiological, biochemical, and molecular factors at different levels of infection. Elevated levels of chitinases pCh2 and pCh11 were reported in the aleurone layer of maize in damaged grains colonized by *A. flavus* (Moore et al., 2004). Hence, breeding to strengthen physical features such as thick testa and chemical barriers such as thick cutin and lignin layers can inhibit *A. flavus* infection and aflatoxin contamination. Similarly, improving the aleurone layer of maize with high chitinase and trypsin inhibitor can reduce aflatoxin accumulation.

### Constitutive and Induced Resistance Mechanisms

Host plant resistance to biotic stresses has been characterized into two categories, i.e., constitutive and induced resistance. Phytoanticipins confer constitutive resistance while phytoalexins contribute to induced resistance (VanEtten et al., 1994). Secondary metabolites are known to be involved in controlling several immune responses, e.g., callose deposition and programed cell death (Piasecka et al., 2015). Phytoanticipins are antimicrobial metabolites (Pedras and Yaya, 2015). For instance, the groundnut plant produces a variety of phenylpropanoids, such as *p*-coumaric acid, caffeic acid, ferulic acid, methoxycinnamic acid, and mucilagin A, a phenylpropanoid-polyketide-isoprenoid. These metabolites have been known to have antifungal activities against both *A. flavus* and *A. parasiticus* (Sobolev et al., 2006). These phenylpropanoids are likely to function as phytoanticipins in specific groundnut plant tissues (Pedras and Yaya, 2015). Phenylalanine ammonia lyase (PAL) which is a precursor of lignin and phytoalexins, has increased rapidly and reached maximum levels in resistant groundnut genotypes than in susceptible ones (Liang et al., 2001). In the case of membrane lipid peroxidation, the level of malondialdehyde (MDA) increased by 8-fold 2–3 days after inoculation (DAI). Moreover, the generation of O_2_^–^, H_2_O_2_, and lipoxygenase (LOX) also increased markedly at the early stage after infection in groundnut (Liang et al., 2002). Resveratrol is an antifungal secondary metabolite or phytoalexin compound found in groundnut seeds (Wang et al., 2015). In resistant genotypes, resveratrol levels increased by 30-fold on the third DAI (Liang et al., 2006). In contrast, the resveratrol level remained unchanged even on the 4-DAI in susceptible genotypes. Plants have several inducible defense responses to pathogens, such as lignification, cell wall cross-linking, phytoalexins, hypersensitive response, production of reactive oxygen species (ROS), and pathogenesis-related (PR) proteins (Liang et al., 2006).

In maize, the first line of defense in response to *A. flavus* results in the activation of expression of transcriptional factors such as WRKY that confer resistance against pathogens (Skriver and Mundy, 1990). WRKY transcription factors were found to be significantly upregulated by *A. flavus* infection in developing maize kernels of resistant maize line TZAR101 (Fountain et al., 2015). ZmWRKY53 is highly expressed in response to a necrotrophic pathogen and also regulates chitinase and peroxidase gene expression. Lignin cross-linking in the cell wall contributes to the resistance to *A. flavus* infection. For instance, less *A. flavus* growth was observed in Mp313E, a maize line that has high cross-linked lignin compared to the susceptible line SC212 (Magbanua et al., 2013). For breeding aflatoxin resistance, the genetic transformation or introgression of resistance genes and transcription factors such as WRKY, PAL, and LOX genes can improve groundnut and maize varieties and reduce the burden of aflatoxin contamination.

## Genomic Regions Controlling Aflatoxin Resistance

Several QTL mapping studies have been performed leading to discovery of genomic regions for aflatoxin resistance in groundnut and maize (Table 1). Each QTL mapping experiment in groundnut has had at least one QTL with phenotypic variation explained (PVE) > 10% and reaching up to >20% in some cases. Interestingly in maize, some QTLs were mapped on same genomic regions in different mapping populations which indicated that there are some genes underlying similar function in different studies (Warburton and Williams, 2014; Parish et al., 2019).

**TABLE 1:** Key bi-parental QTL mapping and GWAS studies for discovery of genomic regions controlling aflatoxin contamination in groundnut and maize.

**Population**	**Trait**	**No. of QTLs/MTAs**	**LOD/*p*-value range**	**PVE% range**	**References**

**Groundnut (*Arachis hypogaea*)**					
***Bi-parental QTL mapping***					
Zhonghua 10 × ICG 12625 (RIL population)	PSII	2	3.1–5.0	8.0–13.0	Yu et al., 2019
	AFB_1_	7	3.1–6.4	7.3–17.9	Yu et al., 2019
	AFB_2_	5	3.5–8.8	8.3–21.0	Yu et al., 2019
Yueyou 92 × Xinhuixiaoli (RIL population)	Resistance to *A. flavus*	2	2.9–10.5	5.2–19.0	W. Zhuang (personal communication)
***Genome-wide association study* (*GWAS*)**					
ICRISAT Reference Set 300	Resistance to *A. flavus*	1	9.68 × 10^−7^	24.7	Pandey et al., 2014

**Maize (*Zea mays***)					

***Bi-parental QTL mapping***					
M53 × RA (F_8__:__9_ RIL population)	Resistance to *A. flavus*	8	2.2–5.4	3.6–9.9	Yin et al., 2014
Mp313E × Va35 (F_2__:__3_ population)	Aflatoxin content	20	2.4–8.0	0.2–21.6	Willcox et al., 2013
Mp715 × T173 (F_2__:__3_ population)	Aflatoxin content	12	1.8–11.5	2.7–18.5	Warburton et al., 2011
NC300 × Mp717 (F_2__:__3_ population)	Aflatoxin content	12	−	1.0–11.0	Warburton et al., 2009
B73 × Mp313E (F_2:3_ population)	Aflatoxin content	13	2.9–7.8	5.0–18.4	Brooks et al., 2005
Tex6 × B73 (BC_1_S_1_)	Aflatoxin content	2	3.8–4.2	16.1–17.8	Paul et al., 2003
Tex6 × B73 (F_2__:__3_)	Aflatoxin content	3	2.5–5.2	6.7–15.1	Paul et al., 2003
RA × M53 (RIL population)	Amount of Aflatoxin (AA)	1 major QTL (*qAA8*)	8.42	18.23	Zhang et al., 2016
		6 epistatic QTLs	5.0–5.4	14.05–22.6	Zhang et al., 2016
B73 × CML322 (F_2_S_5_) RIL population	Afl, ICS, IFS, KSP, and SSP	10	2.6–6.2	6.0–16.0	Mideros et al., 2014
B73o2/o2 × CML161 RIL population	Aflatoxin accumulation	9	3.0–4.0	8.0–11.0	Mayfield et al., 2011
B73o2/o2 × CML161 RIL population	Aflatoxin accumulation	9	2.7–3.9	7.8–11.3	Bello, 2007
***Genome-wide association study* (*GWAS*)**					
Maize inbred lines (346 line)	Aflatoxin resistance	6	5.1–5.5	4.8–6.1	Farfan et al., 2015
Inbred lines (300 line)	Resistance to aflatoxin accumulation (RAA)	107	9.8 × 10^–6^ to 2.9 × 10^–10^	5.4–16.0	Warburton et al., 2015
Maize inbred lines (437 lines)	Amount of aflatoxin (AA)	3	1.1 × 10^−8^ to 2.1 × 10^−7^	6.7–10.4	Zhang et al., 2016
	Resistance to *A. flavus* infection (RAI)	22	3.7 × 10^−22^ to 8.7 × 10^−6^	6.4–26.8	Zhang et al., 2016
Maize inbred lines (287 lines)	Grain aflatoxin levels	298 Maize Cyc pathways	2.9 × 10^–10^ to 1.0	6.4 × 10^–14^ to 0.3	Tang et al., 2015

*BC_1_S_1_, selfed backcross population; PSII, percent seed infection index; AFB_1_, aflatoxin B_1_; AFB_2_, aflatoxin B_2_; IVSC, *in vitro* seed colonization; RIL, recombinant inbred lines; Chr, chromosome; LOD, logarithm of odds; ICS, infection on silk tissue; IFS, infection frequency on silk tissue; KSP, sporulation on developing kernels; SSP, sporulation on silk tissue; Afl, aflatoxin accumulation.*

In groundnut, very few genetic mapping studies have been reported for aflatoxin resistance. Individual QTLs were identified for AFB_1_, AFB_2_, and (percent seed infection index; PSII) using a recombinant inbred line (RIL) population Zhonghua 10 × ICG 12625 by Yu et al. (2019). The study identified two QTLs for PSII, one on chromosome A03 with 8.0% PVE and another on chromosome A10 with 13.0% PVE. Seven QTLs were identified for AFB_1_ (Aflatoxin B_1_) resistance, of which two major QTLs were detected on chromosomes A07 and B06 with 17.9 and 16.3% PVE, respectively. Similarly, five QTLs were identified for resistance to AFB_2_, of which chromosomes A07, B05, B06, and B07 recorded higher PVEs of 12.2, 11.1, 21.0, and 14.5% PVE, respectively. Two consistent QTLs for AFB_1_ (Aflatoxin B_1_) and AFB_2_ (Aflatoxin B_2_) and one for PSII were identified (Yu et al., 2019). Genetic mapping using a groundnut RIL population Yueyou 92 × Xinhuixiaoli for IVSC identified two major QTLs on chromosomes A03 and B04 with LOD of 10.5 and 2.9 and 19.0 and 5.1% PVE, respectively (W. Zhuang, personal communication). Similarly, genome-wide association studies using a groundnut reference set identified a marker associated with IVSC explaining 24.7% PVE (Pandey et al., 2014). One groundnut MAGIC population using eight genotypes possessing resistance to *Aspergillus* infection and reduced aflatoxin accumulation has been developed at ICRISAT for genetic dissection of component traits.

In the case of maize, major effect QTLs were identified in crosses Tex6 × B73 (F_2__:__3_) and Tex6 × B73 (BC_1_S_1_) on chromosomes 3, 4, 5, and 10 with 6.7–17.8% PVE (Paul et al., 2003). Another study (Brooks et al., 2005) conducted in F_2__:__3_-derived maize populations reported two major effect QTLs for aflatoxin resistance in B73 × Mp313E population that were significant across environments. Other studies in maize have identified one stable QTL in NC300 × Mp717 population which was stable across years. Warburton et al. (2009), three major effect QTLs explaining PVE ranging from 12.1–21.6% in Mp313E × Va35 population (Willcox et al., 2013); small effect QTLs in M53 × Mo17 population (Yin et al., 2014), and single QTL explaining 18.5% PVE in Mp715 × T173 population (Warburton et al., 2011). Similarly, QTL for log aflatoxin accumulations were detected on chromosomes 1, 3, 4, and 9, explaining a total of 17% PVE; while QTL for aflatoxin were detected on chromosomes 3, 4, and 8, explaining a total of 15% PVE in RIL population B73o2/o2 × CML161 (Mayfield et al., 2011). In fact, the same population (B73o2/o2 × CML161) was used earlier (Bello, 2007). QTLs affecting aflatoxin from both parents; however, the favorable alleles for the QTL detected by Bello (2007) were derived mainly from CML161 (Mayfield et al., 2011). In earlier aflatoxin QTL studies, Brooks et al. (2005) evaluated their germplasm in four environments, Paul et al. (2003) used two environments, and Warburton et al. (2009) used four environments. All these studies reported few significant QTLs detected in more than one environment. Warburton et al. (2009) reported the most, with one QTL present in all four environments and one QTL detected in two environments. However, Mayfield et al. (2011) reported three QTLs one on each of chromosomes 1, 4, and 9, across multiple years and environments. In another study by using the B73 × CML322 population, ten QTLs with 6.0–16.0% PVE were found using two QTL mapping methods, six of which were located on the same chromosome segments using both approaches (Mideros et al., 2014). By using various sources of near-isogenic lines (NILs) for selected loci, the resistance QTL located in bin 4.08 was confirmed using a NIL pair. Furthermore, the meta-analysis of QTLs using data from 12 populations indicated that the QTL in bin 4.08 has been reported in four mapping populations. The study showed that the largest-effect QTL, located in bin 4.08, is a good candidate for further characterization and use.

In addition to bi-parental QTL mapping studies, many diverse association panels have been used for genome-wide association study (GWAS) leading to the identification of markers/genomic regions for aflatoxin resistance in maize. For instance, Farfan et al. (2015) identified 6 MTAs for aflatoxin resistance with 4.79–6.06% PVE. In another study (Warburton et al., 2015), GWAS analysis using 300 maize inbred lines identified 107 SNPs associated with aflatoxin accumulation in one or more environments in the association panel. Similarly, in another study using an association panel of 437 maize inbred lines, Zhang et al. (2016) identified 3 MTAs for AA and 22 MTAs for resistance to *A. flavus* infection (RAI). In a comprehensive GWAS analysis undertaken by Tang et al. (2015), 298 maize Cyc pathways were reported to be associated with resistance mechanisms, 17 of the pathways reported high enrichment scores of false discovery rate (FDR) < 0.2, of which the jasmonic acid biosynthesis pathway seems to be a major one for aflatoxin resistance. While these studies are informative, comprehensive efforts are required to perform high resolution GWAS in maize and especially in groundnut so that candidate genomic regions/genes can be identified and validated for breeding applications.

## Molecular Basis of Aflatoxin Resistance Mechanisms

### Identification of Resistance-Associated Proteins

Proteomics approaches have identified several plant proteins involved in host-pathogen interaction and in controlling resistance to fungal invasion and toxin production in both groundnut and maize. For instance, in groundnut, a 2D-based proteomics study identified pathways/proteins including resistance-associated proteins (RAPs) which were associated with pre-harvest aflatoxin resistance under drought stress conditions (Wang et al., 2010). That study highlighted the role of iso Ara-h3, oxalate oxidase, PII protein, trypsin inhibitor, SAP domain-containing protein, CDK1, L-ascorbate peroxidase, RIO kinase, and heat shock proteins in reducing aflatoxin accumulation at pre-harvest aflatoxin resistance. Later, Wang et al. (2012) identified several RAPs in groundnut which were key controllers of pathways such as immune signaling, PAMP perception, cell wall responses, and detoxification. The study on effect of H_2_O_2_-derived oxidative stress on *A. flavus* isolates discovered a sub-set of genes that control fungus pathogenicity, mycelial development, and manage ROS production (Fountain et al., 2018).

In maize, several proteomic approaches have been used to understand the molecular mechanisms involved in host-pathogen interaction and resistance to AP. For instance, RIP and zeamatin were present in higher concentrations in germinating maize kernels and led to decreased aflatoxin levels in susceptible maize kernels and thereby inhibited the growth of *A. flavus* under imbibed conditions (Guo et al., 1997). A similar study has indicated the importance of fungal cell wall degrading enzymes, particularly isoforms of beta-l,3-glucanase and chitinase, which are induced in maturing kernels in response to *A. flavus* infection and also in maturing uninfected kernels (Lozovaya et al., 1998; Ji et al., 2000). Importantly, antifungal proteins chitinase and zeamatin appear to be associated with the host first and second layer of resistance (Guo et al., 1997), and their constitutive expression in maize can provide resistance against *A. flavus*. Grains of resistant maize genotypes can accumulate inhibitory proteins such as 22 and 28kDa which restrict the growth of the fungus as they are associated key resistant proteins like PR-5 thaumatin-like proteins and zeamatin (Huang et al., 1997; Moore et al., 2004). In another study, the proteome analysis of resistant maize genotypes identified a constitutive expression of 14-kDa trypsin inhibitor that can cause spore rupture and abnormal hyphal development in *A. flavus* (Chen et al., 1998). Also, the trypsin inhibitor produced by maize can inhibit fungal-amylase activity that limits pathogen access to the host food resource (starch) which in turn restrict fungus mycelial growth and sclerotia development (Woloshuk et al., 1997; Chen et al., 1998, 1999).

A proteomic examination of maize seeds has identified several groups of proteins associated with the embryo and endosperm that were significantly upregulated upon *A. flavus* infection. These proteins were grouped into four categories: storage proteins, water stress-related proteins, PR proteins, and antifungal proteins (Chen et al., 2002, 2004b, 2006, 2007, 2012). Storage proteins globulin 1 and 2, water stress responsive related proteins WSI18, aldose reductase, late embryogenesis abundant (LEA; LEA3 and LEA14) and heat stress related proteins (HSP16.9) impart kernel resistance (Chen et al., 2002). Further, glyoxalase I (GLX-I; EC 4.4.1.5), a stress-related protein, directly controls methylglyoxal levels, an aflatoxin inducing substrate, thereby contributing to lower aflatoxin levels in resistant maize genotypes (Chen et al., 2004b). The RAP involves maize PR-10, which exhibits ribonucleolytic and antifungal activities (Chen et al., 2006, 2007); and the genes of encoding PR proteins are usually highly expressed in resistant genotypes (Chen et al., 2007). A United States–Africa collaborative project identified resistant maize inbred lines (Menkir et al., 2006, 2008; Meseka et al., 2018). The project reported the development of 52 BC_1_S_4_ lines from crosses between five African maize inbreds and five temperate aflatoxin-resistant lines followed by the identification of RAPs related to antifungal, stress-related, storage or regulatory protein categories (Chen et al., 2012). Resistant inbred lines of maize are known to express higher levels of chitinase and proteins associated with phenylpropanoid metabolism pathways (Peethambaran et al., 2009; Pechanova et al., 2011).

Using multiple approaches in groundnut and maize have led to the identification of several moderate/low/high resistant lines for *A. flav*us infection and reduced aflatoxin contamination. These advances have facilitated the development of aflatoxin-resistant transgenic groundnut (Sharma et al., 2018) and maize (Thakare et al., 2017); and it is expected that in the coming years, farmers may have access to superior and aflatoxin-resistant varieties. However, the release of transgenic cultivars is dependent on their acceptance by regulators in the target countries. To date, the use of transgenic maize is accepted only in South Africa and Sudan in Africa. A summary of different proteomic studies in maize and groundnut is provided in Table 2. Cumulatively, these studies enhance our knowledge of target proteins in order to identify protein encoding resistance genes in response to aflatoxin contamination in these crops.

**TABLE 2:** List of key proteins and their functions associated with resistance to aflatoxin contamination in groundnut and maize.

**RAPs**	**Function**	**References**
**Groundnut**		
Oxalate oxidase	Seed storage protein	Wang et al., 2010
Trypsin inhibitor	Antifungal compound	
SAP domain-containing protein	Abiotic stress tolerance protein	
L-ascorbate peroxidase	Regulates antioxidant metabolism	
Iso Ara-h3	Seed storage protein	
Heat shock protein precursor	Regulates heat shock factors	
LRR receptor serine/threonine kinase	PAMPs perception	Wang et al., 2012
Protein phosphatase 2A regulatory B subunit	Dephosphorylation	
Pentatricopeptide repeat-containing protein	RNA stabilization	
Esterase_lipase	Lipid metabolism	
Cytochrome P450	Degrades toxins	
**Maize**		
Zeamatin	Antimicrobial, fungicide	Guo et al., 1997; Huang et al., 1997; Chen et al., 2002
Ribosome-inactivating protein (RIP)	Protein synthesis inhibitor	Guo et al., 1997
Chitinase	Hydrolytic enzymes that degrade chitin	Guo et al., 1997; Ji et al., 2000; Chen et al., 2002
Glucanase	Destroys cell wall of fungi	Guo et al., 1997
Beta-1,3-glucanase	PR-2 family protein, antifungal	Lozovaya et al., 1998; Ji et al., 2000
PR-5 thaumatin-like protein	PR protein	Huang et al., 1997
Globulin-1,2	Seed storage proteins	Chen et al., 2001, 2002, 2006, 2012
Endochitinase	Degrades chitin molecule at random point	Huang et al., 1997
14-kDa trypsin inhibitor	Spores rupture and cause abnormal hyphal development	Chen et al., 1998, 1999
LEA3,14	Stress responsive proteins	Chen et al., 2002, 2006, 2012
WSI18 and aldose reductase	Osmo-stress responsive and oxidative stress responsive proteins	Chen et al., 2002
HSP16.9 (Heat stress related)	Stress responsive protein	Chen et al., 2002
Glyoxalase I	Controls methylglyoxal level as it stimulates the expression of *aflR*, an aflatoxin regulatory gene	Chen et al., 2004a
PR-10	Disease resistance	Chen et al., 2006
Stress-related-peroxiredoxin antioxidant (PER1)	Antioxidants proteins that protect against oxygen species	
Heat shock proteins (HSP17.2)	Stress responsive proteins	
Antifungal trypsin inhibitor protein (TI)	Inhibits *A. flavus* growth	
Cold-regulated protein (COR)	Inhibits germination of *A. flavus* conidia and mycelial growth	Chen et al., 2006, 2012
Superoxide dismutase	Enhances oxidative stress tolerance	Chen et al., 2012
Peroxiredoxin	Enhances oxidative stress tolerance	
Cupindomain-containing proteins	Seed storage protein	
Putative lipid transfer protein	Stress responsive	
Eukaryotic translation initiation factor 5A	Plays a role in plant growth and development	
Abiotic stress responsive proteins	PR protein and stress responsive	Peethambaran et al., 2009
PRm3 chitinase	Fungal cell wall degradation and stress resistance	
Chitinase 1	Defense mechanism in response to biotic stress	
Chitinase A	Suppresses fungal growth	
Phenylpropanoid metabolism	Secondary metabolite production	Pechanova et al., 2011

### Identification of Candidate Genes

Functional genomics provides new insights into a wide number of candidate genes associated with resistance to aflatoxin contamination in both groundnut and maize (Table 3). In the case of groundnut, transcriptomics studies have identified candidate genes, pathways, and the regulatory networks for the three resistance mechanisms of aflatoxin accumulation (IVSC, PAC, and AP). Earlier efforts to identify resistance/differentially expressed genes in groundnut were based on EST or microarray-based techniques (Luo et al., 2005; Guo et al., 2011; Wang et al., 2013). The gene expression profiling approach was deployed by Luo et al. (2005) in A13 drought-tolerant and pre-harvest aflatoxin-resistant groundnut genotypes in which a cDNA microarray containing 384 unigenes was selected from two cDNA libraries. Overall, the microarray-based screening approach identified defense responsive (Kunitz-type trypsin inhibitor, auxin repressed protein, cystatin-like protein), signaling component (ethylene-responsive protein, calcium-binding protein), ion-proton transporter (aquaporin 1), stress proteins, and secondary metabolites (lipoxygenase 1) resistance genes in groundnut in response to *A. parasiticus* infection under drought stress (Luo et al., 2005).

**TABLE 3:** A summary of some transcriptomics studies to identify candidate genes involved in aflatoxin contamination in groundnut and maize.

**Candidate genes**	**Functions of candidate genes**	**References**
**Groundnut**		
Seed maturation protein LEA 4	Stress responsive protein	Guo et al., 2008
Serine protease inhibitor	Involved in inflammatory responses	
Cu/Zn superoxide dismutase II	Antioxidant defensive protein	
Serine protease inhibitor	Involved in inflammatory responses	
Lipoxygenase	Regulates jasmonic acid signaling pathway	Guo et al., 2011
Proline-rich protein	Stress responsive protein	
Cupin//Oxalate oxidase	Seed storage protein	
LEA-protein 2	Stress responsive protein	
Brassinosteroid Insensitive 1-associated Receptor kinase 1	Defense response	Wang et al., 2013
3-ketoacyl-CoA synthase	Fatty acid biosynthetic process	
Em protein	Stress responsive	
TIR	Defense response	
Defensin	Defense response	
Mitogen-activated protein kinase	Signaling cascade gene	Wang et al., 2016
PR proteins	Disease resistance	
Nucleotide-binding site-leucine-rich repeat proteins	PAMPs perception	
Polygalacturonase inhibitor proteins	Inhibit polygalactouronase produced by the fungal pathogen	
Abscisic acid insensitive5	Participates in ABA signaling pathway	Clevenger et al., 2016
BLH1	Modulates seed development	
Respiratory burst oxidase homolog	Regulates numerous plant cell responses	
13S-lipoxygenases	Lipid metabolism	
PR-2	Disease resistance in plants	
Deoxy-chalcone synthase	Synthesizes phytoalexins	
Resveratrol synthase	Biosynthesis stilbene type-phytoalexins	Nayak et al., 2017
Chalcone synthase	Involved in the flavonoid biosynthesis pathway	
Epoxide hydrolase	Detoxification of reactive epoxide	
Receptor-like kinases	Cell wall signaling	
9s-LOX	Lipid metabolism	
WRKY genes	Transcriptional regulators; regulates plant development	Korani et al., 2018
Toll/Interleukin-1 receptor-nucleotide-binding site leucine-rich repeat (TIR-NBS-LRR)	Defense responsive	
α-linolenic acid metabolism	Lipid metabolism	
Hevamine-A	Defense protein	Zhao et al., 2019
PR proteins	Disease resistance	
Chitinase	Hydrolytic enzymes that degrade chitin	
**Maize**		
Kunitz-type trypsin inhibitor	Serine protease inhibitor activity	Luo et al., 2005
Auxin repressed protein	Regulates growth and disease resistance	
Cystatin-like protein	Defense mechanism	
Lipoxygenase 1	Regulates the jasmonic acid pathway	
Ion-proton transporter (Aquaporin 1),	Accelerates oxidative stress and cell signaling	
Glutathione S-transferase	Antioxidant	
Heat shock protein	Defense mechanism; regulates heat shock factors	
PR protein 1	Disease resistance	
ADP glucose pyrophosphorylase	Starch metabolism	Luo et al., 2009
1-acyl-glycerol-3-phosphate acyltransferase	Lipid metabolism	
Lipoxygenase	Regulates the jasmonic acid pathway	
Oleosin 17	Oil body formation and storage protein	
Abscisic acid inducible gene	Defense-related genes	
Chalcone synthase C2	Involved in the flavonoid biosynthesis pathway	
Glutathione transferase	Antioxidant gene	Luo et al., 2011
Leucine-rich repeat-like protein	Biotic stress-related gene	
ABI3-interacting protein 2	A transcription factor of the abscisic acid signal transduction pathway that plays a role in seed development	
Beta-1,3-glucanase	Classified in PR-2 family of PR proteins, antifungal	
Zeamatin-like protein	Antimicrobial, fungicide	
PR genes	PR genes	
Phosphoglycerate dehydratase 1	Plays a role in catalysis	Luo et al., 2010
Heat shock protein 90	Signal transduction and stress responsive	
Glycine−rich protein	Stress responsive and signaling	
Cytochrome P450	Degrades toxins	
Ethylene-responsive element binding factor	Regulates jasmonic acid signaling pathway	
9-oxylipins	Suppresses aflatoxin biosynthesis pathway	Fountain et al., 2013
Lipoxygenase-3 (LOX3)	Regulates jasmonic acid signaling pathway	
PR proteins	Disease resistance	
NUP85-like genes	Transports RNA, R-proteins and macromolecules from the nucleus to the cytoplasm	Kelley et al., 2012
Heat shock protein (HSP101)	Molecular chaperone protein	
Molecular chaperones	Plays a role in protein folding	
Cinnamoyl-CoA	Synthesizes lignin compounds	
PR-4	Antifungal proteins play a role in pathogenicity	Dhakal et al., 2017
Leucine-rich repeat family protein	Highly conserved region for disease resistance genes	
DEAD-box RNA helicase	Defense-related signaling	
Fructose-1,6-bisphosphatase	Carbohydrate metabolism	
Plant receptor protein kinases (RPK)	Senses pathogen signals and accelerates defense	
Cysteine proteinase inhibitor	Stress responsive	
PR-1, PR-4, PR-5, PR-10	Disease resistance-related genes	
CC-NBS-LRR	Conserves disease resistance genes	Shu et al., 2017
LRR-RLK	Conserves disease resistance genes	
Thaumatin- like protein	Regulates host defense mechanism	
Chitinase	Hydrolytic enzymes that degrade chitin	

To understand the molecular mechanism of host-mediated resistance, a separate study was conducted in *Aspergillus* resistant (GT-C20) and susceptible (Tifrunner) genotypes of groundnut which identified 52 highly and 126 moderately expressed genes (Guo et al., 2011). This study reported several important genes including lipoxygenase, lea-protein 2, proline-rich protein, cupin//Oxalate oxidase, among others, in response to *A. flavus* infection. Some studies have suggested the possible involvement of LOX pathway in the production of jasmonic acid which plays hormone-like regulatory and defense-related roles in plants (Royo et al., 1996; Kolomiets et al., 2001; Yan et al., 2013; Ogunola et al., 2017).

Studies have reported that LOX genes also play a major role in plant defense mechanisms, growth, and developmental processes (Kolomiets et al., 2001, 2018; Gao et al., 2008; Park and Kolomiets, 2010). In this emerging field, more investigations are needed on host-pathogen cross-talk communication that fungi use to exploit the plant host in order to meet their biological needs (Christensen and Kolomiets, 2011). Some LOX genes have been shown to play an important role in plant defense resistance and in mediating fungal colonization and toxin production (Battilani et al., 2018).

A microarray study representing 36,158 unigenes was used to identify genes associated with aflatoxin resistance in groundnut (Wang et al., 2013), providing insights into the co-regulation of multiple pathways such as host defensive responses including carbohydrate biosynthesis/metabolism, transmembrane transport, coenzyme A biosynthesis, oxidation-reduction, proteolysis metabolism, etc., during aflatoxin resistance. Modern approaches such as RNA-seq have been used to identify host resistance associated pathways in different crops including maize and groundnut. For instance, in case of groundnut, an integrated IVSC and RNA-seq approach that analyzed the four different stages of infected seed samples from J11 (resistant) and JL24 (susceptible) identified 4,445 differentially expressed unigenes (DEGs) that were involved in multiple pathways such as defense-related, PR or metabolic pathway targeting genes provided a more solid understanding of cross-talk between host-pathogen interactions (Nayak et al., 2017).

Likewise, an RNA-seq-based approach was deployed in groundnut to identify genes that confer resistance during PAC (Clevenger et al., 2016). The study was able to associate the role of abscisic acid (ABA) signaling pathway during drought stress-induced aflatoxin contamination and/or PAC, and also revealed the role of genes from the fatty acid metabolism, cell wall restructuring and morphology, sugar metabolism and nitrogen metabolism pathways during *A. flavus* contamination in soil. Recently, Zhao et al. (2019) suggested the role of hevamine-A protein in groundnut during PAC resistance. Hevamine-A protein is an enzyme with chitinase activity that is also coordinated with PR proteins and can directly inhibit the growth of *A. flavus* (Zhao et al., 2019).

Post-harvest aflatoxin contamination can take place during drying, storage or transportation due to increase in humidity and/or insect damage, thereby promoting *A. flavus* infection. To understand the post-harvest resistance mechanism, Wang et al. (2016) performed global transcriptome profiling in the grains of resistant (Zhonghua 6) and susceptible (Zhonghua 12) genotypes of groundnut and identified 30,143 DEGs, of which 842 were defense-related genes, including mitogen-activated protein kinase, PR proteins, leucine-rich repeat receptor-like kinases transcription factors, nucleotide-binding site-leucine-rich repeat proteins, polygalacturonase inhibitor proteins, and ADP-ribosylation factors in response to AP by *A. flavus*. A recent study by Korani et al. (2018) provides new insights into post-harvest resistance mechanism in response to *A. flavus* infection by comparing the seed transcriptome of resistant (ICG 1471) and susceptible (Florida-07) groundnut cultivars. The study identified 4,272 DEGs and showed the importance of WRKY TFs, heat shock proteins and TIR-NBS-LRR in providing resistance. Further, this study also showed the altered expression of genes associated with protein processing in the endoplasmic reticulum, spliceosome mediated protein degradation and α-linolenic acid metabolism.

In maize, gene expression analysis of inbred line Tex6 identified 8,497 positive array spots including genes related to disease resistance (chitinase, zeamatin-like protein, endochitinase B precursor, PR-1;4;5), stress responsive (heat shock proteins, auxin responsive factor-1, D-type cyclin), ROS scavenger (glutathione S-transferase, superoxide dismutase), and defense-related genes, as well as storage protein genes and lipid metabolism genes (Luo et al., 2009). Further, Luo et al. (2010) have shown that jasmonate and abscisic acid biosynthetic and signaling pathways play crucial roles in drought-induced *A. flavus* infection and accumulation of aflatoxin in maize. The transcriptomic study of resistant maize (Eyl25) with susceptible (Eyl31) lines identified 530 DEGs including defense-related genes; beta-1,3-glucanase, zeamatin-like protein, trypsin inhibitor, and PR genes (Luo et al., 2011). Fountain et al. (2013) have highlighted the role of WRKY TFs in conferring resistance to *Aspergillus* infection and subsequently in reduced PAC in maize genotype. The transcriptomic study of maize kernels in two resistant inbred lines (Mp313E and Mp04:86) and two susceptible inbred lines (Va35 and B73) under artificial inoculation conditions identified NUP85-like genes in resistance (Kelley et al., 2012). The NUP85-like protein is a major part of nuclear pore complex (NPCs) and is involved in the transportation of RNA, R-proteins, and other macromolecules from the nucleus to the cytoplasm (Cheng et al., 2009; Garcia and Parker, 2009). A few more genes like heat shock protein (HSP101), metallothionein-like protein (MTLP), lecithin cholesterol acyltransferase (LCAT)-like gene, Prenylated Rab PRA1 proteins, molecular chaperones, and detoxification proteins were found to be highly expressed in resistant maize inbred line Mp313E. Some genes including a nuclease-phosphatase domain superfamily protein, a cinnamoyl-CoA, a heat shock protein HSP18a, and few significantly mapped genes like lysine-rich RNA binding domains, large and small ribosomal units had significantly higher expression in susceptible line Va35 than in resistant line Mp313E (Kelley et al., 2012).

Climate change has a devastating impact on mycotoxin production and fungal infection. Functional genomics tools have shown the impact of elevated CO_2_ levels on *aflR* gene (an aflatoxin biosynthetic regulatory gene) in *A. flavus* (Gilbert et al., 2016). A cDNA library of Mp715 (resistant inbred) and B73 (susceptible inbred) was designed to differentiate expression patterns for aflatoxin accumulation in maize, and those cDNA clones were mapped onto the maize genome by *in silico* mapping (Dhakal et al., 2017). This study identified 267 unigenes related to stress tolerance, metabolism, disease resistance, PR-4, and leucine-rich repeat family protein. A comparative study of maize kernels infected with *A. flavus* and *F. verticillioides* identified several candidate genes such as PR-1, 10,4,5,10.1; chitinase, CC-NBS-LRR, LRR-RLK, and Thaumatin-like proteins that showed temporal expression patterns during infection/stress (Shu et al., 2017). Several environmental/external factors affect the expression of transcripts, thus influencing the colonization of *A. flavus* and subsequently toxin production. For instance, the antifungal fumigant benzenamine affects aflatoxin biosynthesis, development, and virulence in *A. flavus* by downregulating the *LeaA* regulatory factor, thus acting as a fumigant against *A. flavus* (Yang et al., 2019).

### Transgenic Approaches for Resistance to *A. flavus* Infection and Aflatoxin Contamination

Several transgenic approaches including expressing protein/enzyme that can reduce fungal infection or degrade the toxin have been deployed in groundnut and maize to mitigate aflatoxin contamination (Table 4). In groundnut, very few reports on transgenic approaches are available substantiating the importance of host genes like *PR* and *defensin* (Xie et al., 2013; Arias et al., 2015). A study (Sharma et al., 2018) has shown that the overexpression of *Medicago* defensin genes- *MsDef1 and MtDef4.2* reduced *Aspergillus* infection as well as AP in susceptible groundnut variety JL 24. The study also demonstrated a host-induced gene silencing (HIGS) mediated silencing of aflatoxin biosynthetic pathway regulatory genes *aflM* and *aflP* to inhibit AP. Notably, both OE−Def and HIGS lines showed remarkably reduced levels of aflatoxin B_1_ ranging from 1 to 20 ppb compared to the wild type cultivar that accumulates up to > 4,000 ppb.

**TABLE 4:** A summary of some overexpression, RNAi and host-induced gene silencing studies in groundnut and maize.

**Gene**	**Source**	**Approach**	**Promoter**	**Outcome**	**References**
**Groundnut**					
*ARAhPR10*	*A. hypogaea*	Overexpression	CaMV35S	Transgenic lines showed both reduced infection and less aflatoxin production	Xie et al., 2013
*aflR; aflS; aflJ; aflep; aflC*/*pksA*/*pksL1, pes1*	*A. flavus*	RNA interference gene silencing technology	CaMV35S	Transgenic lines showed up to 100% reduction in aflatoxin content	Arias et al., 2015
*MsDef1; MtDef4*	*M. sativa; M. truncatula*	Overexpression	FMV35S	OE*-Def* lines showed a significant reduction in aflatoxin content (up to 99%) HIGS lines showed a significant reduction in aflatoxin content (up to 99.9%)	Sharma et al., 2018
*aflM; aflP*	*A. flavus*	Host-induced-gene silencing approach	CaMV35S		
**Maize**					
*ZmPR10*	*Z. mays*	RNA interference gene silencing technology	CaMV35S promoter	Downregulation of *PR-10* caused increased susceptibility and aflatoxin contamination	Chen et al., 2010
*Thanatin*	*Podisus maculiventris*	Heterologous expression	Ubiquitin-1 promoter	Cloning of thanatin (an antimicrobial synthetic peptide) improved resistance and reduced aflatoxin content (up to 68%)	Schubert et al., 2015
*aflR*	*A. flavus*	Host-induced-gene silencing approach	Ubiquitin promoter	Transgenic lines showed up to 14-fold less aflatoxin concentration compared to the wild type	Masanga et al., 2015
*aflC*	*A. flavus*	RNA interference	γ-zein endosperm-specific promoter	Transgenic lines showed up to 100% reduction in aflatoxin content	Thakare et al., 2017
*ZmPRms*	*Z. mays*	RNA interference based gene silencing	Zein promoter	Downregulation of *ZmPRms* gene caused increased susceptibility and aflatoxin contamination	Majumdar et al., 2017
*AGM182*	*Tachypleus tridentatus*	Overexpression	Ubiquitin-1 promoter	Overexpression of *AGM182* (an antimicrobial peptide) caused suppression of *A. flavus* growth and subsequently aflatoxin production (up to 98%)	Rajasekaran et al., 2018

Various studies on maize provide insights into using transgenic approaches and the knowledge of precise engineering strategies to improve food safety. A key approach is RNA interference (RNAi), a technology that limits the transcription of a target gene. This approach has been deployed to silence RAP genes (PR-10, GLXI, TI) in maize to identify the key role of RAPs in host resistance mechanism against *A. flavus* infection (Chen et al., 2004a, 2010). RNAi Pr10 silencing construct was introduced in maize plants showing increased susceptibility to *A. flavus* colonization and aflatoxin accumulation (Chen et al., 2010). Notably, PR-10 was involved in enhancing plant stress tolerance and severe suppression of their PR protein encoding genes drastically increased susceptibility to *A. flavus* infection (Xie et al., 2010; Majumdar et al., 2017). Recently, *aflC* and *aflR* genes were targeted that encode the enzyme in *Aspergillus* aflatoxin biosynthetic pathway to develop aflatoxin-free transgenic kernels (Masanga et al., 2015; Thakare et al., 2017). Also, thanatin, a growth inhibitor of *A. flavus*, was overexpressed in maize, reducing aflatoxin contamination and increasing resistance by three to four-fold resistance (Schubert et al., 2015).

In a recent study, expression analyses of polyamine (PA) metabolism/transport genes during *A. flavus*-maize interaction showed significant increase in the expression of arginine decarboxylase (*Adc*) and *S*-adenosylmethionine decarboxylase (*Samdc*) genes in the maize host and PA uptake transporters in the fungus (Majumdar et al., 2018). This study suggested that future studies targeting spermidine biosynthesis in *A. flavus*, using RNAi-based host-induced gene silencing approaches, may be an effective strategy to reduce aflatoxin contamination in maize and possibly in other susceptible crops. In contrary, Gressel and Polturak (2018) report that RNAi technology can’t help post-harvest AP as it may have only limited utility when the grain has been dried. However, the dormant state of seeds is usually alleviated during post-harvest storage conditions or under low moisture conditions and cannot accelerate the production of hpRNAs/siRNAs (Majumdar et al., 2017). Even in the post-transcriptional state, RNAi negatively regulates gene expression and does not produce any protein or enzyme in the host plant (Majumdar et al., 2017). Fakhoury and Woloshuk (1999) produced a mutant strain (101) of *A. flavus* which was defective in the α-amylase activity. The α-amylase enzyme is crucial in *A. flavus* as it is involved in the degradation of the host’s carbohydrate reservoir which is an essential energy source for fungus growth and reproduction, as well as AP. Therefore, an α-amylase inhibitor protein (AILP) that inhibits α-amylase activity was expressed in the host; this reduced fungus growth and subsequent AP (Fakhoury and Woloshuk, 2001; see Chen et al., 2015). Recently, a transgenic maize line expressing *AGM182* which encodes a tachyplesin1-derived synthetic peptide (an antimicrobial peptide) was developed that exhibited reduced fungal growth and a significant reduction in aflatoxin level (76–98%) compared to the control (Rajasekaran et al., 2018). Characterization of these candidate genes through a transgenic approach would be important in safeguarding food commodities.

### Managing Aflatoxin Contamination: Similarities Between Groundnut and Maize

Pre- and post-harvest management strategies largely predict the extent to which *Aspergillus* fungi invade seeds and exacerbate AP (Hell et al., 2008). Most post-harvest management practices like rapid drying of groundnut in-shell and maize ears coupled with appropriate storage conditions are crucial for reducing infection and toxin accumulation. During initiation stage, host-pathogen interactions occur in the cell wall where NBS-LRR receptors, oxylipins, and elicitors play an important role. This is followed by a change in ion flux across the plasma membrane and the activation of a number of genes that lead to changes in the plant’s cell wall. It activates various PR-related proteins, phytoalexins-like compounds and TFs which play an important role in defense mechanism. In addition, at the environmental level, PAC is largely exacerbated by drought stress and insect damage in groundnut and maize (Guo et al., 2008; Hell et al., 2008). Attempts to characterize resistance due to the physical barriers suggested that pod shell may serve as a barrier to *A. flavus* infection when the kernels are stored in-shell in the case of groundnut (Liang et al., 2006; Nigam et al., 2009). Similarly, in maize, a tight husk and non-upright ear act as a barrier to the entry of spores and keep the ear dryer, resulting in an unfavorable environment for fungal growth (Warburton and Williams, 2014). Such physical barriers are considered non-desirable traits since they pose serious challenges while threshing or dehulling.

In groundnut and maize, cross-talk communication between the pathogen and host plant is the first critical step toward the rapid activation of defense mechanisms in host plants. Functional and biological composition of resistance mechanisms in maize and groundnut using integrated approaches have led to the elucidation of the roles of several genes, PR-10, chitinase, 14-kDa trypsin inhibitor, zeatin and beta-1,3-glucanase, lipoxygenase, ROS, and stress responsive proteins (such as late embryogenesis abundant protein (LEA14), catalase, glutathione S-transferase, superoxide dismutase, heat shock proteins) which play a vital role in regulating resistance and in cross-kingdom interactions between host plants and *Aspergillus* species in groundnut (Luo et al., 2005; Chadha and Das, 2006; Liang et al., 2006; Wang et al., 2010; Guo et al., 2011; Kumari et al., 2011; Nayak et al., 2017) and maize (Guo et al., 1997; Chen et al., 1998, 1999, 2001, 2002, 2004b, 2006, 2007, 2012; Lozovaya et al., 1998; Ji et al., 2000; Moore et al., 2004; Magbanua et al., 2007; Pechanova et al., 2011; Pegoraro et al., 2011; Roze et al., 2013; Fountain et al., 2014, 2016; Hawkins et al., 2015; Ogunola et al., 2017).

## Metabolomics Under *A. flavus* Infection and Aflatoxin Resistance

Metabolomics is an emerging field that represents the complete set of metabolites in a biological cell, tissue, organ or organism. It provides an instantaneous snapshot of the “physiological state” of an organism (Ramalingam et al., 2015; Kumar R. et al., 2017). Metabolites are small molecules that are directly involved in growth, development, and reproduction processes.

To understand the aflatoxin resistance mechanism at the metabolite level, some metabolome studies in response to *A. flavus* infection have been conducted in maize. For instance, metabolome profile under *A. flavus* infection showed significant induction and higher expression of polyamine (PA) biosynthesis genes in maize-resistant lines TZAR102, MI82 than in susceptible line SC212. Higher expression of spermidine (Spd), spermine (Spm), and diamine putrescine (Put) along with their increased catabolism in the resistant lines than in the susceptible line indicate that polyamines play an important role in *A. flavus* resistance (Majumdar et al., 2019). In addition, higher concentrations of amino acids such as glutamate (Glu), glutamine (Gln), and γ-aminobutyric acid in susceptible maize line SC212 showed that these amino acids favor *A. flavus* infection. In a similar study by Falade et al. (2018), metabolites were analyzed at R3 (milk), R4 (dough), and R5 (dent) stages of cob development under *A. flavus* infection (4 doses). The study showed that grain colonization decreases with increasing kernel maturity from milk-, dough-, and dent-stage kernels, with approximately 100%, 60%, and 30% colonization, respectively. However, aflatoxin levels increase with increased doses at dough and dent stages. This shows that initial stages of cob development (milk and dough) are more susceptible than the maturity stage (Falade et al., 2018). A study on aflatoxin accumulation in grains of 120 maize hybrids showed that higher concentrations of beta-carotene (BC), beta-cryptoxanthin (BCX), and total provitamin A had significantly less aflatoxin accumulation compared to that in hybrids with lower carotenoid concentration. Hence, breeding for increased carotenoid concentration can increase aflatoxin resistance in maize to help combat aflatoxin contamination as well as malnutrition (Suwarno et al., 2019). In short, metabolites significantly influence *A. flavus* infection and can be used as biomarkers for screening resistant and susceptible maize genotypes.

## Molecular Biology of *A. flavus* for Aflatoxin Production and Resistance

**FIGURE 2:**
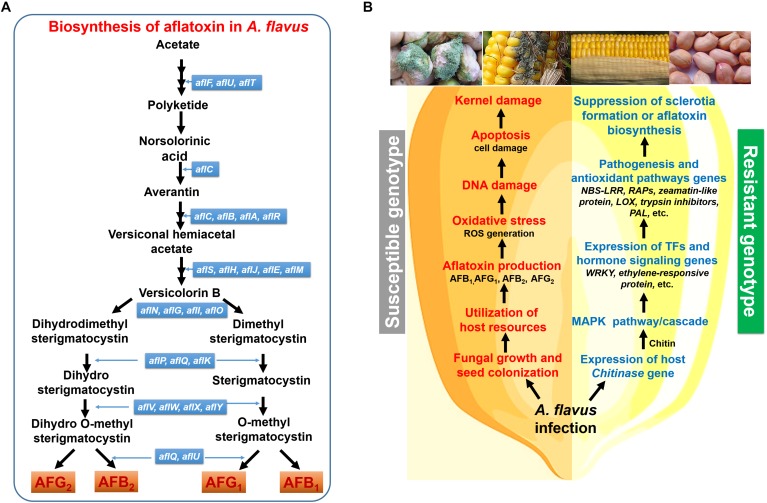
A simplified representation of the aflatoxin biosynthesis pathway and the defense response mechanism in groundnut or maize. **(A)** Aflatoxin biosynthesis in *A. flavus*; **(B)** the aflatoxin biosynthesis pathway involve multiple genes which co-express together for the formation of toxin secondary metabolites. In the susceptible genotype infection leads to the *A. flavus* seed colonization and production of aflatoxin which causes suppression of host defense mechanism results in ROS generation and DNA damage causing cell death (apoptosis). In contrast, in resistant genotypes infection causes induction of host defense mechanism that include MAPK pathway which induces WRKY TF expression which is a key regulator of pathogenesis and antioxidant related genes involved in the suppression of aflatoxin biosynthesis pathway or detoxification of toxin.

The genome of the toxigenic strain of *A. flavus* contains ∼12,000 genes involved in the synthesis of secondary metabolites, with more than 56 gene clusters contributing to the production of secondary metabolites, including aflatoxin (Rokas et al., 2007). The aflatoxin biosynthesis gene cluster includes 25 genes spanning approximately 70 kb of DNA (Yu et al., 2004). The aflatoxin gene cluster resides on chromosome 3, next to the telomeric region comprising of pathway-specific regulatory genes as well as surrounded by four sugar-utilization genes at the distal end (Yu et al., 2000). Some regulatory genes (e.g., *aflR* and *aflS*) are reported to be essential for the production of aflatoxin after infection, and they work in conjunction with several other regulators/factors such as *VelB*/*VeA*/*LaeA* complex, *CreA* transcription factor, among others. While the *aflR* gene encodes a DNA binding Zn-cluster protein that binds to DNA binding-domains of aflatoxin pathway genes, *aflS* is an aflatoxin pathway-specific regulatory gene required to mediate *aflR* transportation to/from the nucleus and assist in *aflR* localization (Figure 2; Ehrlich et al., 2012).

*Aspergillus flavus* can hijack the host machinery to facilitate the uptake of resources required for AP. For instance, the fungus requires the spermidine synthase (a polyamine biosynthetic gene) for AP and can utilize the host substrate to enhance polyamine (PA) biosynthesis and AP (Majumdar et al., 2018). In susceptible maize kernel, the expression of the PA biosynthetic/metabolism genes *S-adenosylmethionine decarboxylase* (*Samdc*) and *arginine decarboxylase* (*Adc*) significantly increased; this was followed by the upregulation of PA transporters in the pathogen (Majumdar et al., 2018). Maize’s hypersensitivity and susceptibility to *A. flavus* involve a gene encoding glycine-rich RNA binding protein 2 which is associated with hormone and pathogen stress (Kelley et al., 2012), through salicylic-mediated defense signal transduction and HR reactions (Naqvi et al., 1998; Singh et al., 2011). The NPCs which transport RNA and other macromolecules are highly expressed in resistant maize cultivars and suppress *A. flavus* infection (Kelley et al., 2012). In Arabidopsis, a defect in *MOS7* (an NPC encoding gene) suppresses the accumulation of R-protein in the nucleus that causes a defect in both basal and systemic acquired resistance and R-protein-mediated immunity (Cheng et al., 2009). The infection induces higher expression of ethylene-responsive protein (ETHRP) in resistant maize cultivars suggesting the role of the ethylene signaling pathway in aflatoxin accumulation resistance. ETHRP is a universal stress protein and a key regulator of stress responses, and confers stress survival (Kelley et al., 2012). Further, fungal infection induces the production of several antifungal proteins such as 14-kDa trypsin inhibitor, 18 kDa ribosome-inactivating-protein, 28, 38 and 100 kDa protein, non-specific lipid transfers proteins, 2 S storage proteins, and zeamatin (Liang et al., 2006). An infection can also induce lipid peroxidation, which facilitates resistance to AP in groundnut (Liang et al., 2002).

*Aspergillus* infection also involves a dynamic network of transcription factors that coordinate the expression of the target biosynthetic genes of the pathogen and the suppression of the host’s immune responses. This may involve the suppression of key gene *WRKY*, a transcription factor that modulates the expression of several genes involved in detoxification of ROS as well as aflatoxin (Korani et al., 2018), including NBS-LRR; its suppression is linked to aggravated accumulation of aflatoxin in plants such as groundnut (Nayak et al., 2017). Further, these TFs are also associated with PR proteins, which play a major role in resistance after infection (Pierpoint et al., 1981; Van Loon, 1985; Szerszen, 1990; Van Loon and Van Strien, 1999). In groundnut, WRKY and other key TFs such as ERF and NAC function in a coordinated fashion (Nayak et al., 2017; Korani et al., 2018); their modulation has a substantial impact on antioxidant biosynthetic, PR proteins, chitinase, and beta-1,3-glucanase genes. Modulation of these TFs in the host severely affects the transcription of ROS detoxifying genes such as catalases, superoxide dismutase, glutathione-S-transferase, and antioxidant biosynthesis genes like resveratrol synthase, PAL, chalcone synthase, chitinase, and beta-1,3-glucanase (Nayak et al., 2017; Korani et al., 2018). These genes protect host plants from oxidative damage, increase the levels of secondary metabolites involved in lignin biosynthesis, and restrict fungal invasion as well as its growth. In resistant groundnut genotypes, the activity of PAL enzyme that catalyzes the metabolism of phenolic compounds such as phytoalexin and lignin precursors, increases significantly (Nayak et al., 2017; Korani et al., 2018).

Resveratrol is a potent phytoalexin induced up to 30-fold in resistant genotypes of groundnut seeds upon infection (Liang et al., 2006). In wild groundnut species, the pod shell and seeds are rich in lignin content that prevents aflatoxin contamination (Guimarães et al., 2012). Notably, in maize, exposure to drought severely reduces PAL enzyme activity and phytoalexin production due to reduced moisture content in the kernel, resulting in fungal invasion and toxin production (Gholizadeh, 2011). Although, studies spanning 15 years have identified several gene clusters regulating host-pathogen interactions and AP, the characterization of individual genes is crucial to design strategies toward mitigation of aflatoxin contamination.

## Challenges and Opportunities

*Aspergillus flavus* infection and subsequent aflatoxin contamination is highly influenced by environmental parameters such as high soil temperature, moisture stress, and relative humidity which often outsmart the low levels of genetic resistance available in groundnut and maize genotypes. This could be one of the key reasons in making this trait very complex and limited progress has been made under field conditions as compared to controlled environment. Even under controlled environmental conditions, most studies are targeted at understanding host-pathogen interactions using a single toxigenic *A. flavus* strain and its interaction with the host (groundnut or maize). However, under field conditions, the reality is different. Often, many species of *Aspergillus* group of fungi such as *A. flavus* and *A. parasiticus* are involved in causing aflatoxin contamination. The population dynamics of toxigenic *Aspergillus* in soils and possible shifts in toxigenic and non-toxigenic strains could be an important area to focus on while studying host-pathogen interactions. Also required is a knowledge of the soil composition of toxigenic *A. flavus* group of fungi and the ambient environment in a crop production region that drives *Aspergillus* population levels and other competing and co-existing pathogens. Similar conditions can be created/simulated under a controlled environment to facilitate the easy adoption and translation of results from laboratory conditions to the field. The lack of consistency in host-pathogen-toxin interactions inhibits the understanding of the precise genetic behavior of resistance in groundnut and maize. Despite a sequencing revolution in the last decade, genetic and gene discovery efforts have not led to solutions to aflatoxin reduction because of inconsistent phenotyping results. Devising novel phenotyping techniques to assay AP at different steps is a way forward. Dissecting components of resistance using known pre-harvest resistant sources of groundnut and maize may be an interesting area of research. In this context, studying the biochemical composition of the seed coat could lead to a better understanding of host-pathogen interactions.

Another key challenge as well as an opportunity would be to understand the impact of soil and its environment on AP. Plants growing in unhealthy soils are bound to be more stressed, and this might increase aflatoxin contamination. While most studies have concentrated on the physical and chemical components of soil, the biological component remains unexplored. An analysis of the phytobiome, the microbial component that surrounds the plant, from the leaves down to the roots, is another emerging area of research. A phytobiome that negatively impacts plant health would influence aflatoxin contamination. Insights into the phytobiomes of groundnut and maize would certainly influence our understanding of host-pathogen interactions, especially in complex traits such as aflatoxin contamination.

## Summary

While discussing the progress made in understanding the resistance mechanisms of aflatoxin contamination in groundnut and maize using multidisciplinary approaches, the paper elaborates on several QTLs, genes, pathways and complex genetic architecture of the target trait. The paper has also reviewed the potential of different approaches in better understanding the complexities of candidate genes identified after the genome sequencing of host and pathogen. Various cultural and biological methods have been reported to prevent/sustainably manage aflatoxin contamination in groundnut and maize. The development of varieties/hybrids or transgenics with resistance to both fungal infection and aflatoxin contamination remains a challenge. To date, aflatoxin management strategies have centered around the use of good agricultural practices during pre-and post-harvest stages, including the use of biocontrol agents (particularly of non-toxigenic strains of *A. flavus*) in countries where they are available to farmers. Omics studies in the last couple of decades provide an array of genetic and genomic resources and expand the knowledge base on *Aspergillus* infection and aflatoxin reduction mechanisms, host-pathogen interactions, toxigenicity of the fungi, mechanism of aflatoxin biosynthesis, and inhibitors targeting the aflatoxin biosynthetic genes. Promising genomics and transgenic approaches have provided complimentary beneficial effects by integrating genes, peptides/antifungal proteins, and even silencing key genes for *Aspergillus* growth and aflatoxin biosynthesis in susceptible varieties to enhance resistance levels. These integrated approaches comprising of functional and structural genomics, together with NGS platform will provide more information on candidate genes to facilitate the development of molecular markers for use in molecular breeding. Conventional and modern breeding tools need to be deployed to develop aflatoxin-resistant maize and groundnut varieties that will lead to food safety, poverty reduction and boosting the industry and market.

## Author Contributions

RV together with MP and PS conceptualized the idea after receiving the invitation from the journal. PS together with SG, AO-B, RK, SP, HS, YL, XN, DH, JF, SN, GM, TR, WZ, BG, BL, PSi, MP, and RB wrote the manuscript. PS, MP, and RV finalized the manuscript.

## Conflict of Interest

The authors declare that the research was conducted in the absence of any commercial or financial relationships that could be construed as a potential conflict of interest.

## References

[B1] Adhikari B. N. Bandyopadhyay R. Cotty P. J. (2016). Degeneration of aflatoxin gene clusters in *Aspergillus flavus* from Africa and North America. *AMB Express* 6:62. 10.1186/s13568-016-0228-6 27576895 PMC5005231

[B2] Agbetiameh D. Ortega-Beltran A. Awuah R. T. Atehnkeng J. Cotty P. J. Bandyopadhyay R. (2018). Prevalence of aflatoxin contamination in maize and groundnut in Ghana: population structure, distribution, and toxigenicity of the causal agents. *Plant Dis.* 102 764–772. 10.1094/PDIS-05-17-0749-RE 30673407 PMC7779968

[B3] Agbetiameh D. Ortega-Beltran A. Awuah R. T. Atehnkeng J. Islam M.-S. Callicott K. A. (2019). Potential of atoxigenic strains of *Aspergillus flavus* associated with maize and groundnut in Ghana as biocontrol agents for aflatoxin management. *Front. Microbiol.* 10:2069 10.3389/fmicb.2019.02069 PMC6743268 31555251

[B4] Alaniz Zanon M. S. Barros G. G. Chulze S. N. (2016). Non-aflatoxigenic *Aspergillus flavus* as potential biocontrol agents to reduce aflatoxin contamination in peanuts harvested in Northern Argentina. *Int. J. Food Microbiol.* 231 63–68. 10.1016/j.ijfoodmicro.2016.05.016 27220011

[B5] Alaniz Zanon M. S. Chiotta M. L. Giaj-Merlera G. Barros G. Chulze S. (2013). Evaluation of potential biocontrol agent for aflatoxin in Argentinean peanuts. *Int. J. Food Microbiol.* 162 220–225. 10.1016/j.ijfoodmicro.2013.01.017 23454811

[B6] Amaike S. Keller N. P. (2011). *Aspergillus flavus*. *Annu. Rev. Phytopathol.* 49 107–133. 10.1146/annurev-phyto-072910-095221 21513456

[B7] Arias R. S. Dang P. M. Sobolev V. S. (2015). RNAi-mediated control of aflatoxins in peanut: method to analyze mycotoxin production and transgene expression in the peanut/*Aspergillus pathosystem*. *J. Vis. Exp.* 106 1–11. 10.3791/53398 26709851 PMC4694054

[B8] Asis R. Barrionuevo D. L. Giorda L. M. Nores M. L. Aldao M. A. (2005). Aflatoxin production in six peanut (*Arachis hypogaea* L.) genotypes infected with *Aspergillus flavus* and *Aspergillus parasiticus*, isolated from peanut production areas of Cordoba Argentina. *J. Agric. Food Chem.* 53 9274–9280. 10.1021/jf051259 16277433

[B9] Atehnkeng J. Donner M. Ojiambo P. S. Ikotun B. Augusto J. Cotty P. J. (2016). Environmental distribution and genetic diversity of vegetative compatibility groups determine biocontrol strategies to mitigate aflatoxin contamination of maize by *Aspergillus flavus*. *Microb. Biotechnol.* 9 75–88. 10.1111/1751-7915.12324 26503309 PMC4720411

[B10] Ayalew A. Kimanya M. Matumba L. Bandyopadhyay R. Menkir A. Cotty P. (2017). “Controlling aflatoxins in maize in Africa: strategies, challenges and opportunities for improvement,” in *Achieving Sustainable Cultivation of Maize. Cultivation Techniques, Pest and Disease Control*, Vol. 2 ed. Watson D., (Cambridge: Burleigh Dodds Science Publishing), 1–24.

[B11] Bandyopadhyay R. Atehnkeng J. Ortega-Beltran A. Akande A. Falade T. D. O. Cotty P. J. (2019). “Ground-truthing” efficacy of biological control for aflatoxin mitigation in farmers’ fields in Nigeria: from field trials to commercial usage. *Front. Microbiol.* 10:2528 10.3389/fmicb.2019.02528 PMC6882503 31824438

[B12] Bandyopadhyay R. Ortega-Beltran A. Akande A. Mutegi C. Atehnkeng J. Kaptoge L. (2016). Biological control of aflatoxins in Africa: current status and potential challenges in the face of climate change. *World Mycotoxin J.* 9 771–789. 10.3920/wmj2016.2130

[B13] Battilani P. Lanubile A. Scala V. Reverberi M. Gregori R. Falavigna C. (2018). Oxylipins from both pathogen and host antagonize jasmonic acid-mediated defence via the 9-lipoxygenase pathway in *Fusarium verticillioides* infection of maize. *Mol Plant Pathol.* 19 2162–2176. 10.1111/mpp.12690 29660236 PMC6638020

[B14] Bello H. T. (2007). *Phenotypic and Genotypic Evaluation of Generations and Recombinant Inbred Lines for Response to Aflatoxin.* Ph.D. dissertation Texas A&M University, College Station, TX.

[B15] Brooks T. D. Williams W. P. Windham G. L. Willcox M. C. Abbas H. K. (2005). Quantitative trait loci contributing resistance to aflatoxin accumulation in maize inbred Mp313E. *Crop Sci.* 45 171–174. 10.1371/journal.pone.0036892 22606305 PMC3351445

[B16] Brown R. Cotty P. Cleveland T. Widstrom N. (1993). Living maize embryo influences accumulation of aflatoxin in maize kernels. *J. Food Prot.* 56 967–971. 10.4315/0362-028X-56.11.967 31113092

[B17] Bryden W. L. (2012). Mycotoxin contamination of the feed supply chain: implications for animal productivity and feed security. *Anim. Feed Sci. Tech.* 173 134–158. 10.1016/j.anifeedsci.2011.12.014

[B18] Busboom K. N. White D. G. (2004). Inheritance of resistance to aflatoxin production and *Aspergillus* ear rot of corn from the cross of inbreds B73 and Oh516. *Phytopathology* 94 1107–1115. 10.1094/PHYTO.2004.94.10.1107 18943800

[B19] Campbell K. W. Hamblin A. M. White D. G. (1997). Inheritance of resistance to aflatoxin production in the cross between corn inbreds B73 and LB31. *Phytopathology* 87 1144–1147. 10.1094/PHYTO.1997.87.11.1144 18945011

[B20] Campbell K. W. White D. G. (1995). Evaluation of corn genotypes for resistance to *Aspergillus* ear rot, kernel infection, and aflatoxin production. *Plant Dis.* 79 1039–1045.

[B21] Chadha P. Das R. H. (2006). A pathogenesis related protein, AhPR10 from peanut: an insight of its mode of antifungal activity. *Planta* 225 213–222. 10.1007/s00425-006-0344-7 16832688

[B22] Chang P. K. Horn B. W. Dorner J. W. (2005). Sequence breakpoints in the aflatoxin biosynthesis gene cluster and flanking regions in nonaflatoxigenic *Aspergillus flavus* isolates. *Fungal Genet. Biol.* 42 914–923. 10.1016/j.fgb.2005.07.004 16154781

[B25] Chen Z. Y. Brown R. L. Cleveland T. E. Damann K. E. (2004a). Investigating the roles of an aflatoxin resistance-associated protein in maize using RNAi. *Phytopathology* 94 S18–S18.

[B23] Chen Z. Y. Brown R. L. Cleveland T. E. Damann K. E. Russin J. S. (2001). Comparison of constitutive and inducible maize kernel proteins of genotypes resistant or susceptible to aflatoxin production. *J. Food Protect.* 64 1785–1792. 10.4315/0362-028x-64.11.1785 11726160

[B24] Chen Z. Y. Brown R. L. Damann K. E. Cleveland T. E. (2002). Identification of unique or elevated levels of kernel proteins in aflatoxin–resistant maize genotypes through proteome analysis. *Phytopathology* 92 1084–1094. 10.1094/PHYTO.2002.92.10.1084 18944219

[B26] Chen Z. Y. Brown R. L. Damann K. E. Cleveland T. E. (2004b). Identification of a maize kernel stress-related protein and its effect on aflatoxin accumulation. *Phytopathology* 94 938–945. 10.1094/PHYTO.2004.94.9.938 18943070

[B27] Chen Z. Y. Brown R. L. Damann K. E. Cleveland T. E. (2007). Identification of maize kernel endosperm proteins associated with resistance to aflatoxin contamination by *Aspergillus flavus*. *Phytopathology* 97 1094–1103. 10.1094/PHYTO-97-9-1094 18944174

[B28] Horn B. W. Ramirez-Prado J. H. Carbone I. (2009b). Sexual reproduction and recombination in the aflatoxin-producing fungus *Aspergillus parasiticus*. *Fungal Genet. Biol.* 46 169–175. 10.1016/j.fgb.2008.11.004 19038353

[B29] Chen Z. Y. Brown R. L. Damann K. E. Cleveland T. E. (2010). PR10 expression in maize and its effect on host resistance against *Aspergillus flavus* infection and aflatoxin production. *Mol. Plant Pathol.* 11 69–81. 10.1111/j.1364-3703.2009.00574.x 20078777 PMC6640484

[B30] Chen Z. Y. Brown R. L. Lax A. R. Guo B. Z. Cleveland T. E. Russin J. S. (1998). Resistance to *Aspergillus flavus* in corn kernels is associated with a 14-kDa protein. *Phytopathology* 88 276–281. 10.1094/PHYTO.1998.88.4.276 18944949

[B31] Chen Z. Y. Brown R. L. Menkir A. Cleveland T. E. (2012). Identification of resistance-associated proteins in closely-related maize lines varying in aflatoxin accumulation. *Mol. Breed.* 30 53–68. 10.1007/s11032-011-9597-3

[B32] Chen Z. Y. Brown R. L. Rajasekaran K. Damann K. E. Cleveland T. E. (2006). Identification of a maize kernel pathogenesis-related protein and evidence for its involvement in resistance to *Aspergillus flavus* infection and aflatoxin production. *Phytopathology* 96 87–95. 10.1094/PHYTO-96-0087 18944208

[B33] Chen Z. Y. Brown R. L. Russin J. S. Lax A. R. Cleveland T. E. (1999). A corn trypsin inhibitor with antifungal activity inhibits *Aspergillus flavus* α-amylase. *Phytopathology* 89 902–907. 10.1094/PHYTO.1999.89.10.902 18944733

[B34] Chen Z. Y. Rajasekaran K. Brown R. L. Sayler R. J. Bhatnagar D. (2015). Discovery and confirmation of genes/proteins associated with maize aflatoxin resistance. *World Mycotoxin J.* 8 211–224. 10.3920/wmj2014.1732

[B35] Cheng Y. T. Germain H. Wiermer M. Bi D. Xu F. García A. V. (2009). Nuclear pore complex component MOS7/Nup88 is required for innate immunity and nuclear accumulation of defense regulators in *Arabidopsis*. *Plant Cell* 21 2503–2516. 10.1105/tpc.108.064519 19700630 PMC2751965

[B36] Christensen S. A. Kolomiets M. V. (2011). The lipid language of plant–fungal interactions. *Fungal Genet. Biol.* 48 4–14. 10.1016/j.fgb.2010.05.005 20519150

[B37] Clevenger J. Marasigan K. Liakos V. Sobolev V. Vellidis G. Holbrook C. (2016). RNA sequencing of contaminated seeds reveals the state of the seed permissive for pre-harvest aflatoxin contamination and points to a potential susceptibility factor. *Toxins* 8 1–18. 27827875 10.3390/toxins8110317 PMC5127114

[B38] Cotty P. J. (1989). Virulence and cultural characteristics of two *Aspergillus flavus* strains pathogenic on cotton. *Phytopathology* 79 808–814.

[B39] Cotty P. J. (2001). “Cotton seed losses and mycotoxins,” in *Compendium of Cotton Diseases. Part 1*, eds Kirkpatrick T. L. Rothrock C. S., (St Paul: APS Press), 9–13.

[B40] Cotty P. J. Antilla L. Wakelyn P. J. (2007). “Competitive exclusion of aflatoxin producers: farmer-driven research and development,” in *Biological Control a Global Perspective*, eds Vincent C. Goettel M. S. Lazarovits G., (Wallingford: Centre for Agriculture and Bioscience International), 241–253. 10.1079/9781845932657.0241

[B41] Cotty P. J. Probst C. Jaime-Garcia R. (2008). “Etiology and management of aflatoxin contamination,” in *Mycotoxins: Detection Methods, Management, Public Health and Agricultural Trade*, (Wallingford: Centre for Agriculture and Bioscience International), 287–299. 10.1079/9781845930820.0287

[B42] Danso J. K. Osekre E. A. Opit G. P. Arthur F. H. Campbell J. F. Mbata G. (2019). Impact of storage structures on moisture content, insect pests and mycotoxin levels of maize in Ghana. *J. Stored Prod. Res.* 81 114–120. 10.1016/j.jspr.2018.11.012

[B43] Danso J. K. Osekre E. A. Opit G. P. Manu N. Armstrong P. Arthur F. H. (2018). Post-harvest insect infestation and mycotoxin levels in maize markets in the Middle Belt of Ghana. *J. Stored Prod. Res.* 77 9–15. 10.1016/j.jspr.2018.02.004

[B44] Darrah L. L. Lillehoj E. B. Zuber M. S. Scott G. E. Thompson D. West D. R. (1987). Inheritance of aflatoxin B1 levels in maize kernels under modified natural inoculation with *Aspergillus flavus*. *Crop Sci.* 27 869–872.

[B45] Dhakal R. Chai C. Karan R. Windham G. L. Williams W. P. Subudhi P. K. (2017). Expression profiling coupled with *in-silico* mapping identifies candidate genes for reducing aflatoxin accumulation in maize. *Front. Plant. Sci.* 8:503. 10.3389/fpls.2017.00503 28428796 PMC5382453

[B46] Diedhiou P. M. Bandyopadhyay R. Atehnkeng J. Ojiambo P. S. (2011). *Aspergillus* colonization and aflatoxin contamination of maize and sesame kernels in two agro–ecological zones in Senegal. *J. Phytopathol.* 159 268–275. 10.1111/j.1439-0434.2010.01761.x

[B47] Dorner J. W. (2004). Biological control of aflatoxin contamination of crops. *J. Toxicol. Toxin Rev.* 23 425–450. 10.1081/txr-200027877

[B48] Dorner J. W. (2009). Development of biocontrol technology to manage aflatoxin contamination in peanuts. *Peanut Sci.* 36 60–67. 10.3146/at07-002.1

[B49] Doster M. A. Cotty P. J. Michailides T. J. (2014). Evaluation of the atoxigenic *Aspergillus flavus* strain AF36 in pistachio orchards. *Plant Dis.* 98 948–956. 10.1094/PDIS-10-13-1053-RE 30708840

[B50] Ehrlich K. Mack B. Wei Q. Li P. Roze L. Dazzo F. (2012). Association with *AflR* in endosomes reveals new functions for *AflJ* in aflatoxin biosynthesis. *Toxins* 4 1582–1600. 10.3390/toxins4121582 23342682 PMC3528264

[B51] Ezekiel C. N. Ortega-Beltran A. Oyedeji E. Atehnkeng J. Kössler P. Tairu F. (2019). Aflatoxin in chili peppers in Nigeria: extent of contamination and control using atoxigenic *Aspergillus flavus* genotypes as biocontrol agents. *Toxins* 11:429. 10.3390/toxins11070429 31336571 PMC6669588

[B52] Fakhoury A. M. Woloshuk C. P. (1999). Amy1, the α-amylase gene of *Aspergillus flavus*: involvement in aflatoxin biosynthesis in maize kernels. *Phytopathology* 89 908–914. 10.1094/PHYTO.1999.89.10.908 18944734

[B53] Fakhoury A. M. Woloshuk C. P. (2001). Inhibition of growth of *Aspergillus flavus* and fungal α-amylases by a lectin-like protein from *Lablab purpureus*. *Mol. Plant Microbe Interact.* 14 955–961. 10.1094/mpmi.2001.14.8.955 11497467

[B54] Falade T. D. O. Chrysanthopoulos P. K. Hodson M. P. Sultanbawa Y. Fletcher M. Darnell R. (2018). Metabolites identified during varied doses of *Aspergillus* species in Z*ea mays* grains, and their correlation with aflatoxin levels. *Toxins* 10:187. 10.3390/toxins10050187 29735944 PMC5983243

[B55] Farfan I. D. De La Fuente GN Murray S. C. Isakeit T. Huang P. C. Warburton M. (2015). Genome Wide Association Study for Drought, Aflatoxin Resistance, and Important Agronomic Traits of Maize Hybrids in the Sub-Tropics. *PLoS One* 10:e0117737. 10.1371/journal.pone.0117737 25714370 PMC4340625

[B56] Florkowski W. J. Kolavalli S. (2013). “Aflatoxin control strategies in the groundnut value chain in Ghana,” in *Proceedings of the IFPRI Ghana Strategy Support Program Working Paper*, (Washington, DC: IFPRI), 33.

[B57] Fountain J. C. Bajaj P. Nayak S. N. Yang L. Pandey M. K. Kumar V. (2016). Responses of *Aspergillus flavus* to oxidative stress are related to fungal development regulator, antioxidant enzyme, and secondary metabolite biosynthetic gene expression. *Front. Microbiol.* 7:2048. 10.3389/fmicb.2016.02048 28066369 PMC5175028

[B58] Fountain J. C. Koh J. Yang L. Pandey M. K. Nayak S. N. Bajaj P. (2018). Proteome analysis of *Aspergillus flavus* isolate-specific responses to oxidative stress in relationship to aflatoxin production capability. *Sci. Rep.* 8:3430. 10.1038/s41598-018-21653-x 29467403 PMC5821837

[B59] Fountain J. C. Raruang Y. Luo M. Brown R. L. Chen Z. Y. (2013). The potential roles of WRKY transcription factors in regulating maize defense responses against *Aspergillus flavus* infection. *Phytopathology* 103:45.

[B60] Fountain J. C. Raruang Y. Luo M. Brown R. L. Guo B. Z. Chen Z. Y. (2015). Potential roles of WRKY transcription factors in regulating host defense responses during *Aspergillus flavus* infection of immature maize kernels. *Physiol. Mol. Plant P.* 89 31–40. 10.1016/j.pmpp.2014.11.005

[B61] Fountain J. C. Scully B. T. Ni X. Kemerait R. C. Lee R. D. Chen Z. Y. (2014). Environmental influences on maize-*Aspergillus flavus* interactions and aflatoxin production. *Front. Microbiol.* 5:40. 10.3389/fmicb.2014.00040 24550905 PMC3913990

[B62] Frisvad J. C. Hubka V. Ezekiel C. N. Hong S. B. Novakova A. Chen A. J. (2019). Taxonomy of *Aspergillus* section *Flavi* and their production of aflatoxins, ochratoxins and other mycotoxins. *Stud. Mycol.* 93 1–63. 10.1016/j.simyco.2018.06.001 30108412 PMC6080641

[B63] Gao X. Isakeit T. Brodhagen M. Keller N. P. Kolomiets M. V. (2008). Maize lipoxygenase ZmLOX3-mediated pathway suppresses seed colonization, production of spores and mycotoxins by *Aspergilli* spp. *Phytopathology* 98 S57–S57.

[B64] Garcia A. V. Parker J. E. (2009). Heaven’s gate: nuclear accessibility and activities of plant immune regulators. *Trends Plant Sci.* 14 479–487. 10.1016/j.tplants.2009.07.004 19716748

[B65] Garrido-Bazan V. Mahuku G. Bibbins-Martinez M. Arroyo-Bacerra A. Villalobos-López M. Á (2018). Dissection of mechanisms of resistance to *Aspergillus flavus* and aflatoxin using tropical maize germplasm. *World Mycotoxin J.* 11 215–224. 10.3920/wmj2017.2219

[B66] Gembeh S. V. Brown R. L. Grimm C. Cleveland T. E. (2001). Identification of chemical components of corn kernel pericarp wax associated with resistance to *Aspergillus flavus* infection and aflatoxin production. *J. Agric. Food Chem.* 49 4635–4641. 10.1021/jf010450q 11600000

[B67] Gholizadeh A. (2011). Effects of drought on the activity of phenylalanine ammonia lyase in the leaves and roots of maize inbreds. *Aust. J. Basic Appl. Sci.* 5 952–956.

[B68] Gilbert M. K. Mack B. M. Wei Q. Bland J. M. Bhatnagar D. Cary J. W. (2016). RNA sequencing of an nsdC mutant reveals global regulation of secondary metabolic gene clusters in *Aspergillus flavus*. *Microbiol. Res.* 182 150–161. 10.1016/j.micres.2015.08.007 26686623

[B69] Gorman D. P. Kang M. S. Cleveland T. Hutchinson R. L. (1992). Combining ability for resistance to field aflatoxin accumulation in maize grain. *Plant Breed.* 109 296–303. 10.1111/j.1439-0523.1992.tb00188.x

[B70] Gqaleni N. Smith J. E. Lacey J. Gettinby G. (1997). Effects of temperature and water activity, and incubation time on production of aflatoxins and cyclopiazonic acid by an isolate of *Aspergillus flavus* in surface agar culture. *Appl. Environ. Microbiol.* 63 1048–1053. 10.1128/aem.63.3.1048-1053.1997 16535539 PMC1389133

[B71] Gressel J. Polturak G. (2018). Suppressing aflatoxin biosynthesis is not a breakthrough if not useful. *Pest Manag. Sci.* 74 17–21. 10.1002/ps.4694 28762637

[B72] Guimarães P. M. Brasileiro A. C. Morgante C. V. Martins A. C. Pappas G. Silva O. B. (2012). Global transcriptome analysis of two wild relatives of peanut under drought and fungi infection. *BMC Genom.* 13:387. 10.1186/1471-2164-13-387 22888963 PMC3496627

[B73] Guo B. Chen X. Dang P. Scully B. T. Liang X. Holbrook C. C. (2008). Peanut gene expression profiling in developing seeds at different reproduction stages during *Aspergillus parasiticus* infection. *BMC Dev. Biol.* 8:12. 10.1186/1471-213X-8-12 18248674 PMC2257936

[B74] Guo B. Fedorova N. D. Chen X. Wan C. H. Wang W. Nierman W. C. (2011). Gene expression profiling and identification of resistance genes to *Aspergillus flavus* infection in peanut through EST and microarray strategies. *Toxins* 3 737–753. 10.3390/toxins3070737 22069737 PMC3202856

[B75] Guo B. Z. Chen Z. Y. Brown R. L. Lax A. R. Cleveland T. E. Russin J. S. (1997). Germination induces accumulation of specific proteins and antifungal activities in corn kernels. *Phytopathology* 87 1174–1178. 10.1094/PHYTO.1997.87.11.1174 18945015

[B76] Hawkins L. K. Mylroie J. E. Oliveira D. A. Smith J. S. Ozkan S. Windham G. L. (2015). Characterization of the maize chitinase genes and their effect on *Aspergillus flavus* and aflatoxin accumulation resistance. *PLoS One* 10:0126185. 10.1371/journal.pone.0126185 26090679 PMC4475072

[B77] Hedayati M. T. Pasqualotto A. C. Warn P. A. Bowyer P. Denning D. W. (2007). *Aspergillus flavus*: human pathogen, allergen and mycotoxin producer. *Microbiol.* 153 1677–1692. 10.1099/mic.0.2007/007641-0 17526826

[B78] Hell K. Fandohan P. Bandyopadhyay R. Kiewnick S. Sikora R. Cotty P. J. (2008). “Pre-and postharvest management of aflatoxin in maize: an African perspective,” in *Mycotoxins: Detection Methods, Management, Public Health and Agricultural Trade*, eds Bandyopadhyay R. Leslie J. F., (Wallingford: CABI), 219–229. 10.1079/9781845930820.0219

[B79] Horn B. W. Moore G. G. Carbone I. (2009a). Sexual reproduction in *Aspergillus flavus*. *Mycologia* 101 423–429. 10.3852/09-011 19537215

[B80] Horn B. W. Sorensen R. B. Lamb M. C. Sobolev V. S. Olarte R. A. Worthington C. J. (2014). Sexual reproduction in *Aspergillus flavus* sclerotia naturally produced in corn. *Phytopathology* 104 75–85. 10.1094/PHYTO-05-13-0129-R 23883157

[B81] Huang Z. White D. G. Payne G. A. (1997). Corn seed proteins inhibitory to *Aspergillus flavus* and aflatoxin biosynthesis. *Phytopathology* 87 622–627. 10.1094/phyto.1997.87.6.622 18945080

[B82] Jaime-Garcia R. Cotty P. J. (2004). *Aspergillus flavus* in soils and corncobs in south Texas: implications for management of aflatoxins in corn-cotton rotations. *Plant Dis.* 88 1366–1371. 10.1094/PDIS.2004.88.12.1366 30795199

[B83] Ji C. Norton R. A. Wicklow D. T. Dowd P. F. (2000). Isoform patterns of chitinase and β-1, 3-glucanase in maturing corn kernels (*Zea mays* L.) associated with *Aspergillus flavus* milk stage infection. *J. Agr. Food Chem.* 48 507–511. 10.1021/jf9905119 10691666

[B84] Kachapulula P. W. Akello J. Bandyopadhyay R. Cotty P. J. (2017). *Aspergillus* section *Flavi* community structure in Zambia influences aflatoxin contamination of maize and groundnut. *Int. J Food Microbiol.* 261 49–56. 10.1016/j.ijfoodmicro.2017.08.014 28915412 PMC5644832

[B85] Kelley R. Y. Williams W. P. Mylroie J. E. Boykin D. L. Harper J. W. Windham G. L. (2012). Identification of maize genes associated with host plant resistance or susceptibility to *Aspergillus flavus* infection and aflatoxin accumulation. *PLoS One* 7:e0036892. 10.1371/journal.pone.0036892 22606305 PMC3351445

[B86] Khan M. A. Asghar M. A. Iqbal J. Ahmed A. Shamsuddin Z. A. (2014). Aflatoxins contamination and prevention in red chillies (*Capsicum annuum* L.) in Pakistan. *Food Addit. Contaminants Part B Surveill.* 7 1–6. 10.1080/19393210.2013.825330 24779970

[B87] Kolomiets M. Battilani P. Borrego E. Reverberi M. Lanubile A. Scala V. (2018). Mycotoxin contamination in maize is controlled by oxylipin signals. *Phytopathology* 108:1.

[B88] Kolomiets M. V. Hannapel D. J. Chen H. Tymeson M. Gladon R. J. (2001). Lipoxygenase is involved in the control of potato tuber development. *Plant Cell* 13 613–626. 10.1105/tpc.13.3.613 11251100 PMC135504

[B89] Korani W. Chu Y. Holbrook C. C. Ozias-Akins P. (2018). Insight into genes regulating postharvest aflatoxin contamination of tetraploid peanut from transcriptional profiling. *Genetic* 209 143–156. 10.1534/genetics.118.300478 29545468 PMC5937179

[B90] Kumar P. Mahato D. K. Kamle M. Mohanta T. K. Kang S. G. (2017). Aflatoxins: a global concern for food safety, human health and their management. *Front. Microbiol.* 7:2170 10.3389/fmicb.2016.02170 PMC5240007 28144235

[B91] Kumar R. Bohra A. Pandey A. K. Pandey M. K. Kumar A. (2017). Metabolomics for plant improvement: status and prospects. *Front. Plant Sci.* 8:1302. 10.3389/fpls.2017.01302 28824660 PMC5545584

[B92] Kumari A. M. Devi P. U. Sucharitha A. (2011). Differential effect of lipoxygenase on aflatoxin production by *Aspergillus* spp. *Int J Plant Pathol.* 4 153–164. 10.3923/ijpp.2011.153.164

[B93] LaPrade J. C. Bartz J. A. Nordan A. J. DeMuynk T. J. (1973). Correlation of peanut seed coat surface wax accumulation with tolerance to colonization by *Aspergillus flavus*. *J. Am. Peanut Res. Educ. Soc.* 5 89–94.

[B94] Liang X. Zhou G. Hong Y. Chen X. Liu H. Li S. (2009). Overview of research progress on peanut (*Arachis hypogaea* L.) host resistance to aflatoxin contamination and genomics at the Guangdong Academy of Agricultural Sciences. *Peanut Sci.* 369 29–34. 10.3146/at07-003.1

[B95] Liang X. A. Luo M. Guo B. Z. (2006). Resistance mechanisms to *Aspergillus flavus* infection and aflatoxin contamination in peanut (*Arachis hypogaea*). *Plant Pathol. J.* 51 115–124. 28973784

[B96] Liang X. Q. Pan R. Z. Zhou G. (2002). Involvement of active oxygen generation and lipid peroxidation in the susceptibility/resistance of peanut seed infected by *Aspergillus flavus*. *Chin. J. Oil Crops* 24 19–23.

[B97] Liang X. Q. Zhou G. Y. Pan R. (2001). Changes of some biochemical substances in peanut seeds under infection of *Aspergillus flavus* and their role in resistance to seed invasion. *Chin. J. Oil Crop* 23 26–30.

[B98] Liang X. Q. Zhou G. Y. Pan R. Z. (2003b). Study on the relationship of wax and cutin layers in peanut seeds and resistance to invasion and aflatoxin production by *Aspergillus flavus*. *J. Trop. Subtrop. Bot.* 11 11–14.

[B99] Liang X. Q. Pan R. C. Zhou G. Y. (2003a). Relationship of trypsin inhibitor in peanut seed and resistance to *Aspergillus flavus* invasion. *Acta Agronom. Sin.* 29 295–299.

[B100] Liu Y. Wu F. (2010). Global burden of aflatoxin-induced hepatocellular carcinoma: a risk assessment. *Environ. Health Persp.* 118 818–824. 10.1289/ehp.0901388 20172840 PMC2898859

[B101] Logrieco A. F. Miller J. D. Eskola M. Krska R. Ayalew A. Bandyopadhyay R. (2018). The mycotox charter: increasing awareness of, and concerted action for, minimizing mycotoxin exposure worldwide. *Toxins* 10:149. 10.3390/toxins10040149 29617309 PMC5923315

[B102] Lozovaya V. V. Waranyuwat A. Widholm J. M. (1998). β-l, 3-glucanase and resistance to *Aspergillus flavus* infection in maize. *Crop Sci.* 38 1255–1260. 10.1111/jipb.12286 25251325

[B103] Luo M. Brown R. L. Chen Z. Y. Cleveland T. E. (2009). Host genes involved in the interaction between *Aspergillus flavus* and maize. *Toxin Rev.* 28 118–128. 10.1080/15569540903089197

[B104] Luo M. Brown R. L. Chen Z. Y. Menkir A. Yu J. Bhatnagar D. (2011). Transcriptional profiles uncover *Aspergillus flavus*-induced resistance in maize kernels. *Toxins* 3 766–786. 10.3390/toxins3070766 22069739 PMC3202853

[B105] Luo M. Liang X. Q. Dang P. Holbrook C. C. Bausher M. G. Lee R. D. (2005). Microarray-based screening of differentially expressed genes in peanut in response to *Aspergillus parasiticus* infection and drought stress. *Plant Sci. J.* 169 695–703. 10.1016/j.plantsci.2005.05.020

[B106] Luo M. Liu J. Lee D. Scully B. T. Guo B. (2010). Monitoring the expression of maize genes in developing kernels under drought stress using oligo-microarray. *J. Integr. Plant Biol.* 52 1059–1074. 10.1111/j.1744-7909.2010.01000.x 21106005

[B107] Magbanua Z. V. De Moraes C. M. Brooks T. D. Williams W. P. Luthe D. S. (2007). Is catalase activity one of the factors associated with maize resistance to *Aspergillus flavus*? *MPMI* 20 697–706. 10.1094/mpmi-20-6-0697 17555277

[B108] Magbanua Z. V. Williams P. W. Luthe D. S. (2013). The maize rachis affects *Aspergillus flavus* spread during ear development. *Maydica* 58 182–188.

[B109] Majumdar R. Lebar M. Mack B. Minocha R. Minocha S. Wientjes C. (2018). The *Aspergillus flavus* Spermidine synthase (*spds*) gene, is required for normal development, aflatoxin production, and pathogenesis during infection of maize kernels. *Front. Plant Sci.* 9:317. 10.3389/fpls.2018.00317 29616053 PMC5870473

[B110] Majumdar R. Minocha R. Lebar M. D. Rajasekaran K. Long S. Carter-Wientjes C. (2019). Contribution of maize polyamine and amino acid metabolism towards resistance against *Aspergillus flavus* infection and aflatoxin production. *Front. Plant Sci.* 10:692. 10.3389/fpls.2019.00692 31178889 PMC6543017

[B111] Majumdar R. Rajasekaran K. Sickler C. Lebar M. Musungu B. M. Fakhoury A. M. (2017). The pathogenesis-related maize seed (PRms) gene plays a role in resistance to *Aspergillus flavus* infection and aflatoxin contamination. *Front. Plant Sci.* 8:1758. 10.3389/fpls.2017.01758 29089952 PMC5651032

[B112] Masanga J. O. Matheka J. M. Omer R. A. Ommeh S. C. Monda E. O. Alakonya A. E. (2015). Downregulation of transcription factor *aflR* in *Aspergillus flavus* confers reduction to aflatoxin accumulation in transgenic maize with alteration of host plant architecture. *Plant Cell Rep.* 34 1379–1387. 10.1007/s00299-015-1794-9 25895735

[B113] Mauro A. Battilani P. Cotty P. J. (2015). Atoxigenic *Aspergillus flavus* endemic to Italy for biocontrol of aflatoxins in maize. *Biocontrol* 60 125–134. 10.1007/s10526-014-9624-5

[B114] Mayfield K. L. Murray S. C. Rooney W. L. Isakeit T. Odvody G. A. (2011). Confirmation of QTL reducing aflatoxin in maize testcrosses. *Crop Sci.* 51 2489–2498. 10.2135/cropsci2011.02.0112

[B115] Mehl H. L. Cotty P. J. (2011). Influence of the host contact sequence on the outcome of competition among *Aspergillus flavus* isolates during host tissue invasion. *Appl. Environ. Microbiol.* 77 1691–1697. 10.1128/AEM.02240-10 21216896 PMC3067303

[B116] Mehl H. L. Jaime R. Callicott K. A. Probst C. Garber N. P. Ortega-Beltran A. (2012). *Aspergillus flavus* diversity on crops and in the environment can be exploited to reduce aflatoxin exposure and improve health. *Ann. NY. Acad. Sci.* 1273 7–17. 10.1111/j.1749-6632.2012.06800.x 23230832

[B117] Menkir A. Brown R. L. Bandyopadhyay R. Chen Z. Y. Cleveland T. E. (2006). A USA–Africa collaborative strategy for identifying, characterizing, and developing maize germplasm with resistance to aflatoxin contamination. *Mycopathologia* 162 225–232. 10.1007/s11046-006-0056-3 16944289

[B118] Menkir A. Brown R. L. Bandyopadhyay R. Cleveland T. E. (2008). Registration of six tropical maize germplasm lines with resistance to aflatoxin contamination. *J. Plant Regist.* 2 246–250. 10.3198/jpr2008.01.0028crg

[B119] Meseka S. Williams W. P. Warburton M. L. Brown R. L. Augusto J. Ortega-Beltran A. (2018). Heterotic affinity and combining ability of exotic maize inbred lines for resistance to aflatoxin accumulation. *Euphytica* 214:184.

[B120] Mideros S. X. Warburton M. L. Jamann T. M. Windham G. L. Williams W. P. Nelson R. J. (2014). Quantitative trait loci influencing mycotoxin contamination of maize: analysis by linkage mapping, characterization of near-isogenic lines, and meta-analysis. *Crop Sci.* 54:127 10.2135/cropsci2013.04.0249

[B121] MoFA, (2011). *Agriculture in Ghana. Facts and figures (2010). Statistics, Research and 691 Information Directorate (SRID).* Accra: MoFA.

[B122] Moore K. G. Price M. S. Boston R. S. Weissinger A. K. Payne G. A. (2004). A chitinase from Tex6 maize kernels inhibits growth of *Aspergillus flavus*. *Phytopathology* 94 82–87. 10.1094/PHYTO.2004.94.1.82 18943823

[B123] Naqvi S. S. Park K. S. Yi S. Y. Lee H. W. Bok S. H. Choi D. (1998). A glycine-rich RNA-binding protein gene is differentially expressed during acute hypersensitive response following Tobacco Mosaic Virus infection in tobacco. *Plant Mol. Biol.* 37 571–576. 9617823 10.1023/a:1006031316476

[B124] Nayak S. N. Agarwal G. Pandey M. K. Sudini H. K. Jayale A. S. Purohit S. (2017). *Aspergillus flavus* infection triggered immune responses and host-pathogen cross-talks in groundnut during in-vitro seed colonization. *Sci. Rep.* 7:9659. 10.1038/s41598-017-09260-8 28851929 PMC5574979

[B125] Nigam S. N. Waliyar F. Aruna R. Reddy S. V. Kumar P. L. Craufurd P. Q. (2009). Breeding peanut for resistance to aflatoxin contamination at ICRISAT. *Peanut Sci.* 36 42–49. 10.3146/at07-008.1

[B126] Ogunola O. F. Hawkins L. K. Mylroie E. Kolomiets M. V. Borrego E. Tang J. D. (2017). Characterization of the maize lipoxygenase gene family in relation to aflatoxin accumulation resistance. *PLoS One* 12:e0181265. 10.1371/journal.pone.0181265 28715485 PMC5513560

[B127] Ortega-Beltran A. Bandyopadhyay R. (2019). Comments on “Trial Summary on the Comparison of Various Non-Aflatoxigenic Strains of *Aspergillus flavus* on Mycotoxin Levels and Yield in Maize” by MS Molo, et al. *Agron. J.* 111 2625–2631.

[B128] Pandey M. K. Kumar R. Pandey A. K. Soni P. Gangurde S. S. Sudini H. K. (2019). Mitigating aflatoxin contamination in groundnut through a combination of genetic resistance and post-harvest management practices. *Toxins* 11:315. 10.3390/toxins11060315 31163657 PMC6628460

[B129] Pandey M. K. Upadhyaya H. D. Rathore A. Vadez V. Sheshshayee M. S. Sriswathi M. (2014). Genome wide association studies for 50 agronomic traits in peanut using the ‘reference set’ comprising 300 genotypes from 48 countries of the semi-arid tropics of the world. *PLoS One* 9:e0105228. 10.1371/journal.pone.0105228 25140620 PMC4139351

[B130] Parish F. Williams W. P. Windham G. L. Shan X. (2019). Differential expression of signaling pathway genes associated with aflatoxin reduction quantitative trait loci in maize (*Zea mays* L.). *Front. Microbiol* 10:2683. 10.3389/fmicb.2019.02683 31849861 PMC6901933

[B131] Park Y. Kolomiets M. (2010). Distinct roles of two 9-lipoxygenase paralogs in the regulation of aflatoxin accumulation in maize seed. *Phytopathology* 6 S96–S97.

[B132] Paul C. Naidoo G. Forbes A. Mikkilineni V. White D. Rocheford T. (2003). Quantitative trait loci for low aflatoxin production in two related maize populations. *Theor. Appl. Genet.* 107 263–270. 10.1007/s00122-003-1241-0 12677406

[B133] Payne G. A. (1998). “Process of contamination by aflatoxin-producing fungi and their impact on crops,” in *Mycotoxin in Agriculture and Food safety*, eds Sinha K. K. Bhatnagar D., (New York, NY: Marcel Decker Inc), 279–300.

[B134] Pechanova O. Pechan T. Williams W. P. Luthe D. S. (2011). Proteomic analysis of the maize rachis: potential roles of constitutive and induced proteins in resistance to *Aspergillus flavus* infection and aflatoxin accumulation. *Proteomics* 11 114–127. 10.1002/pmic.201000368 21182199

[B135] Pedras M. S. C. Yaya E. E. (2015). Plant chemical defenses: are all constitutive antimicrobial metabolites phytoanticipins? *Nat. Prod. Commun.* 10 209–218. 25920246

[B136] Peethambaran B. Hawkins L. Windham G. L. Williams W. P. Luthe D. S. (2009). Anti-fungal activity of maize silk proteins and role of chitinases in *Aspergillus flavus* resistance. *Toxin Rev.* 29 27–39. 10.3109/15569540903402874

[B137] Pegoraro C. Mertz L. M. da Maia L. C. Rombaldi C. V. de Oliveira A. C. (2011). Importance of heat shock proteins in maize. *JCSB* 14 85–95.

[B138] Piasecka A. Jedrzejczak-Rey N. Bednarek P. (2015). Secondary metabolites in plant innate immunity: conserved function of divergent chemicals. *New Phytol.* 206 948–964. 10.1111/nph.13325 25659829

[B139] Pierpoint W. S. Robinson N. P. Leason M. B. (1981). The pathogenesis-related proteins of tobacco: their induction by viruses in intact plants and their induction by chemicals in detached leaves. *Physiol. Plant Pathol.* 19 85–97.

[B140] Pildain M. B. Frisvad J. C. Vaamonde G. Cabral D. Varga J. Samson R. A. (2008). Two novel aflatoxin-producing *Aspergillus* species from Argentinean peanuts. *Int. J. Syst. Evol. Microbiol.* 58 725–735. 10.1099/ijs.0.65123-0 18319485

[B141] Pitt J. Manthong C. Siriacha P. Chotechaunmanirat S. Markwell P. (2015). Studies on the biocontrol of aflatoxin in maize in Thailand. *Biocontrol Sci. Techn.* 25 1070–1091. 10.1080/09583157.2015.1028893

[B142] Probst C. Bandyopadhyay R. Cotty P. J. (2014). Diversity of aflatoxin-producing fungi and their impact on food safety in sub-Saharan Africa. *Int. J. Food Microbiol.* 174 113–122. 10.1016/j.ijfoodmicro.2013.12.010 24480188

[B143] Rajasekaran K. Sayler R. J. Sickler C. M. Majumdar R. Jaynes J. M. Cary J. W. (2018). Control of *Aspergillus flavus* growth and aflatoxin production in transgenic maize kernels expressing a tachyplesin-derived synthetic peptide. AGM182. *Plant Sci.* 270 150–156. 10.1016/j.plantsci.2018.02.006 29576068

[B144] Ramalingam A. Kudapa H. Pazhamala L. T. Weckwerth W. Varshney R. K. (2015). Proteomics and metabolomics: two emerging areas for legume improvement. *Front. Plant Sci.* 6:1116. 10.3389/fpls.2015.01116 26734026 PMC4689856

[B145] Razzaghi-Abyaneh M. ed. (2013). *Aflatoxins: Recent Advances and Future Prospects.* Norderstedt: BoD–Books on Demand.

[B146] Rokas A. Payne G. Fedorova N. D. Baker S. E. Machida M. Yu J. (2007). What can comparative genomics tell us about species concepts in the genus *Aspergillus*? *Stud. Mycol.* 59 11–17. 10.3114/sim.2007.59.02 18490942 PMC2275189

[B147] Royo J. Vancanneyt G. Pérez A. G. Sanz C. Störmann K. Rosahl S. (1996). Characterization of three potato lipoxygenases with distinct enzymatic activities and different organ-specific and wound-regulated expression patterns. *J Biol Chem.* 271 21012–21019. 10.1074/jbc.271.35.21012 8702864

[B148] Roze L. V. Hong S. Y. Linz J. E. (2013). Aflatoxin biosynthesis: current frontiers. *Annu. Rev. Food Sci.* 4 293–311. 10.1146/annurev-food-083012-123702 23244396

[B149] Russin J. S. Guo B. Z. Tubujika K. M. Brown R. L. Cleveland T. E. Widstrom N. W. (1997). Comparison of kernel wax from corn genotypes resistant or susceptible to *Aspergillus flavus*. *Phytopathology* 87 529–533. 10.1094/phyto.1997.87.5.529 18945108

[B150] Samson R. A. (1992). Current taxonomic schemes of genus *Aspergillus* and its teleomorphs. *Biotechnolgy* 23:355. 1504606

[B151] Samson R. A. Hoekstra E. S. Van Oorschot C. A. (1981). *Introduction to Food-Borne Fungi. Centraalbureau voor Schimmelcultures.* Available at: https://www.worldcat.org/title/introduction-to-food-borne-fungi/oclc/680002633 (accessed September 10, 2019).

[B152] Sanders T. H. Mixon A. C. (1979). Effect of peanut tannin on percent seed colonization and in vitro growth by *Aspergillus parasiticus*. *Mycopathologia* 66 169–173. 10.1007/bf00683966 440404

[B153] Sarma U. P. Bhetaria P. J. Devi P. Varma A. (2017). Aflatoxins: implications on health. *Indian J. Clin. Biochem.* 32 124–133. 10.1007/s12291-017-0649-2 28428686 PMC5382086

[B154] Schreurs F. Bandyopadhyay R. Kooyman C. Ortega-Beltran A. Akande A. Konlambigue M. (2019). *Commercial Products Promoting Plant Health in African Agriculture.* Cambridge: Burleigh Dodds Science Publishing Limited.

[B155] Schubert M. Houdelet M. Kogel K. H. Fischer R. Schillberg S. Nölke G. (2015). Thanatin confers partial resistance against aflatoxigenic fungi in maize (*Zea mays*). *Transgenic Res.* 24 885–895. 10.1007/s11248-015-9888-2 26071308

[B156] Senghor L. Ortega-Beltran A. Atehnkeng J. Callicott K. Cotty P. Bandyopadhyay R. (2019). The atoxigenic biocontrol product Aflasafe SN01 is a valuable tool to mitigate aflatoxin contamination of both maize and groundnut cultivated in Senegal. *Plant Dis.* 104 510–520. 10.1094/PDIS-03-19-0575-RE 31790640

[B157] Sharma K. K. Pothana A. Prasad K. Shah D. Kaur J. Bhatnagar D. (2018). Peanuts that keep aflatoxin at bay: a threshold that matters. *Plant Biotechnol. J.* 16 1024–1033. 10.1111/pbi.12846 28973784 PMC5902767

[B158] Shu X. Livingston D. P. III Woloshuk C. P. Payne G. A. (2017). Comparative histological and transcriptional analysis of maize kernels infected with *Aspergillus flavus* and *Fusarium verticillioides*. *Front. Plant Sci.* 8:2075. 10.3389/fpls.2017.02075 29270183 PMC5723656

[B159] Singh U. Deb D. Singh A. Grover A. (2011). Glycine-rich RNA binding protein of *Oryza sativa* inhibits growth of M15 *E. coli cells*. *BMC Res.* 4:18. 10.1186/1756-0500-4-18 21269485 PMC3040685

[B160] Skriver K. Mundy J. (1990). Gene expression in response to abscisic acid osmotic stress. *Plant Cell* 2:503 10.2307/3869112 PMC159906 2152172

[B161] Sobolev V. S. Potter T. L. Horn B. W. (2006). Prelylated stilbenes from peanut root mucilage. *Phytochem. Anal.* 17 312–322. 10.1002/pca.920 17019932

[B162] Sudini H. Ranga Rao G. V. Gowda C. L. L. Chandrika R. Margam V. Rathore A. (2015). Purdue Improved Crop Storage (PICS) bags for safe storage of groundnuts. *J. Stored Prod. Res.* 64 133–138. 10.1016/j.jspr.2014.09.002

[B163] Suneja K. (2019). *India Ropes in Global Institution to Curb Contamination in Spice Shipments.* Geneva: World Trade Organization.

[B164] Suwarno W. B. Hannok P. Palacios-Rojas N. Windham G. Crossa J. Pixley K. V. (2019). Provitamin A carotenoids in grain reduce aflatoxin contamination of maize while combating vitamin A deficiency. *Front. Plant Sci.* 10:30. 10.3389/fpls.2019.00030 30778360 PMC6369730

[B165] Szerszen J. B. (1990). Change of isozymes of *Aspergillus* group infected peanut cotyledon from plants grown under drought stress. *APRES* 21:23.

[B166] Taber R. A. Pettit R. E. Benedict C. R. Dieckert J. W. Kertring D. L. (1973). Comparison of *Aspergillus flavus* tolerant and susceptible lines. I. Light microscope investigation. *Am. Peanut Res. Edu. Assoc.* 5:206.

[B167] Tang J. D. Perkins A. Williams W. P. Warburton M. L. (2015). Using genome-wide associations to identify metabolic pathways involved in maize aflatoxin accumulation resistance. *BMC Genomics* 16:673. 10.1186/s12864-015-1874-9 26334534 PMC4558830

[B168] Thakare D. Zhang J. Wing R. A. Cotty P. J. Schmidt M. A. (2017). Aflatoxin-free transgenic maize using host-induced gene silencing. *Sci. Adv.* 3:e1602382. 10.1126/sciadv.1602382 28345051 PMC5345927

[B169] Turner R. B. Linday D. L. Davis D. D. Bishop R. D. (1975). Isolation and identification of 5, 7-dimethoxyisoflavone, an inhibitor of *Aspergillus flavus* from peanut. *Mycopathologia* 57 39–40. 10.1007/bf00431177 813148

[B170] Upadhyaya H. D. Nigam S. N. Thakur R. P. (2002). “Genetic enhancement for resistance to aflatoxin contamination in groundnut,” in *Proceedings of the Seventh ICRISAT Regional Groundnut Meeting for Western and Central Africa, 6–8 Dec. 2000, Cotonou, Benin. Patancheru 502 324*, (Andhra Pradesh: International Crops Research Institute for the Semi-Arid Tropics), 29–36.

[B171] Van Loon L. C. (1985). Pathogenesis-related proteins. *Plant Mol. Biol.* 4 111–116. 24310747 10.1007/BF02418757

[B172] Van Loon L. C. Van Strien E. A. (1999). The families of pathogenesis-related proteins, their activities, and comparative analysis of PR-1 type proteins. *Physiol. Mol. Plant Pathol.* 55 85–97. 10.1006/pmpp.1999.0213

[B173] VanEtten H. D. Mansfield J. W. Bailey J. A. Farmer E. E. (1994). Two classes of plant antibiotics: phytoalexins versus “phytoanticipins.”. *Plant Cell* 6 1191–1192. 10.1105/tpc.6.9.1191 12244269 PMC160512

[B174] Walker S. Jaime R. Kagot V. Probst C. (2018). Comparative effects of hermetic and traditional storage devices on maize grain: mycotoxin development, insect infestation and grain quality. *J. Stored Prod. Res.* 77 34–44. 10.1016/j.jspr.2018.02.002

[B175] Wang H. Lei Y. Wan L. Yan L. Lv J. Dai X. (2016). Comparative transcript profiling of resistant and susceptible peanut post-harvest seeds in response to aflatoxin production by *Aspergillus flavus*. *BMC Plant Biol.* 1:54. 10.1186/s12870-016-0738-z 26922489 PMC4769821

[B176] Wang H. Lei Y. Yan L. Cheng K. Dai X. Wan L. (2015). Deep sequencing analysis of transcriptomes in *Aspergillus flavus* in response to resveratrol. *BMC Microbiol.* 15:182. 10.1186/s12866-015-0513-6 26420172 PMC4589122

[B177] Wang T. Chen X. Li H. Liu H. Hong Y. Yang Q. (2013). Transcriptome identification of the resistance-associated genes (RAGs) to *Aspergillus flavus* infection in pre-harvested peanut (*Arachis hypogaea*). *Funct. Plant Biol.* 40 292–303. 10.1071/FP12143 32481108

[B178] Wang T. Zhang E. Chen X. Li L. Liang X. (2010). Identification of seed proteins associated with resistance to pre-harvested aflatoxin contamination in peanut (*Arachis hypogaea* L). *BMC Plant Biol.* 10:267. 10.1186/1471-2229-10-267 21118527 PMC3095339

[B179] Wang Z. Yan S. Liu C. Chen F. Wang T. (2012). Proteomic analysis reveals an aflatoxin-triggered immune response in cotyledons of *Arachis hypogaea* infected with *Aspergillus flavus*. *J. Proteome Res.* 11 2739–2753. 10.1021/pr201105d 22424419

[B180] Warburton M. L. Brooks T. D. Krakowsky M. D. Shan X. Windham G. L. Williams W. P. (2009). Identification and mapping of new sources of resistance to aflatoxin accumulation in maize. *Crop Sci.* 49 1403–1408. 10.2135/cropsci2008.12.0696

[B181] Warburton M. L. Tang J. D. Windham G. L. Hawkins L. K. Murray S. C. Xu W. (2015). Genome-wide association mapping of *Aspergillus flavus* and aflatoxin accumulation resistance in maize. *Crop Sci.* 55 1857–1867. 27598199

[B182] Warburton M. L. Williams W. P. (2014). Aflatoxin resistance in maize: what have we learned lately? *Adv. Bot.* 2014:352831 10.1155/2014/352831

[B183] Warburton M. L. Brooks T. D. Windham G. L. Williams W. P. (2011). Identification of novel QTL contributing resistance to aflatoxin accumulation in maize. *Mol. Breeding* 27 491–499. 10.1007/s11032-010-9446-9

[B184] Wicklow D. T. Wilson D. M. Nelson T. C. (1993). Survival of *Aspergillus flavus* sclerotia and conidia buried in soil in Illinois or Georgia. *Phytopathology* 83 1141–1147.

[B185] Wild C. P. Gong Y. Y. (2010). Mycotoxins and human disease: a largely ignored global health issue. *Carcinogenesis* 31 71–82. 10.1093/carcin/bgp264 19875698 PMC2802673

[B186] Willcox M. C. Davis G. L. Warburton M. L. Windham G. L. Abbas H. K. Betrán J. (2013). Confirming quantitative trait loci for aflatoxin resistance from Mp313E in different genetic backgrounds. *Mol. Breed.* 32 15–26. 10.1007/s11032-012-9821-9

[B187] Williams W. P. Krakowsky M. D. Scully B. T. Brown R. L. Menkir A. Warburton M. L. (2015). Identifying and developing maize germplasm with resistance to accumulation of aflatoxins. *World Mycotoxin J.* 8 193–209. 10.3920/wmj2014.1751 16944289

[B188] Woloshuk C. P. Cavaletto J. R. Cleveland T. E. (1997). Inducers of aflatoxin biosynthesis from colonized maize kernels are generated by an amylase activity from *Aspergillus flavus*. *Phytopathology* 87 164–169. 10.1094/phyto.1997.87.2.164 18945137

[B189] Xie C. Wen S. Liu H. Chen X. Li H. Hong Y. (2013). Overexpression of ARAhPR10, a member of the PR10 family, decreases levels of *Aspergillus flavus* infection in peanut seeds. *Am. J. Plant Sci.* 4:602 10.4236/ajps.2013.43079

[B190] Xie Y. R. Chen Z. Y. Brown R. L. Bhatnagar D. (2010). Expression and functional characterization of two pathogenesis-related protein 10 genes from *Zea mays*. *J. Plant Physiol.* 167 121–130. 10.1016/j.jplph.2009.07.004 19682768

[B191] Yan Y. Borrego E. Kolomiets M. V. (2013). *Jasmonate Biosynthesis, Perception and Function in Plant Development and Stress Responses Lipid metabolism.* London: InTech, 393–442.

[B192] Yang M. Lu L. Li S. Zhang J. Li Z. Wu S. (2019). Transcriptomic insights into benzenamine effects on the development, aflatoxin biosynthesis, and virulence of *Aspergillus flavus*. *Toxins* 11:70. 10.3390/toxins11020070 30691218 PMC6410012

[B193] Yin Z. Wang Y. Wu F. Gu X. Bian Y. Wang Y. (2014). Quantitative trait locus mapping of resistance to *Aspergillus flavus* infection using a recombinant inbred line population in maize. *Mol. Breed.* 33 39–49. 10.1007/s11032-013-9932-y

[B194] Yu B. Huai D. Huang L. Kang Y. Ren X. Chen Y. (2019). Identification of genomic regions and diagnostic markers for resistance to aflatoxin contamination in peanut (*Arachis hypogaea* L.). *BMC Genet.* 20:32. 10.1186/s12863-019-0734-z 30866805 PMC6417274

[B195] Yu J. Chang P. K. Bhatnagar D. Cleveland T. E. (2000). Genes encoding cytochrome P450 and monooxygenase enzymes define one end of the aflatoxin pathway gene cluster in *Aspergillus parasiticus*. *Appl. Microbiol. Biotechnol.* 53 583–590. 10.1007/s002530051660 10855719

[B196] Yu J. Chang P. K. Ehrlich K. C. Cary J. W. Bhatnagar D. Cleveland T. E. (2004). Clustered pathway genes in aflatoxin biosynthesis. *Appl. Environ. Microbiol.* 70 1253–1262. 10.1128/aem.70.3.1253-1262.2004 15006741 PMC368384

[B197] Zhang Y. Cui M. Zhang J. Zhang L. Li C. Kan X. (2016). Confirmation and fine mapping of a major QTL for aflatoxin resistance in maize using a combination of linkage and association mapping. *Toxins* 8:258. 10.3390/toxins8090258 27598199 PMC5037484

[B198] Zhao X. Li C. Yan C. Wang J. Yuan C. Zhang H. (2019). Transcriptome and proteome analyses of resistant preharvest peanut seed coat in response to *Aspergillus flavus* infection. *Electron. J. Biotechn.* 39 82–90. 10.1016/j.ejbt.2019.03.003

